# Versatile application of magnesium-related bone implants in the treatment of bone defects

**DOI:** 10.1016/j.mtbio.2025.101635

**Published:** 2025-03-05

**Authors:** Mijia Tao, Yutao Cui, Shicai Sun, Yan Zhang, Jianli Ge, Wen Yin, Peng Li, Yanbing Wang

**Affiliations:** a Traumatic Orthopedics, The Second Hospital of Jilin University, Changchun, 130041, PR China; b The Third Affiliated Hospital of Changchun University of Chinese Medicine, PR China

**Keywords:** Magnesium, Bone implants, Bone defect, Bone repair, Mechanism

## Abstract

Magnesium-related bone implants have garnered significant attention in the treatment of bone defects. The applications of magnesium in promoting bone repair mainly include degradable magnesium-based scaffolds owing to its special physical properties and composite materials modified by magnesium ions because of its biological activity. Although numerous studies have confirmed the unique application advantages and efficacy of magnesium in promoting bone repair, some obvious shortcomings persist, including the rapid degradation of magnesium-based scaffolds. In this review, the deficiencies of magnesium and its alloys in the construction of orthopedic implants and their key influencing factors were summarized; furthermore, some advanced improvement schemes were summarized and analyzed. Additionally, the application strategies of magnesium-modified bone implants are summarized and discussed. Lastly, this review incorporates the latest research and discoveries on magnesium in orthopedic science, comprehensively exploring the mechanism of magnesium's role in the complex microenvironment of bone defects from multiple dimensions. This paper provides a comprehensive summary and analysis of cutting-edge concepts in the design and development of magnesium-based bone implants, considering various perspectives such as the physical properties and biological functions of magnesium.

## Highlights

• Magnesium-related bone implants enhance bone repair through degradable scaffolds.
• Advanced schemes improve magnesium scaffold stability and therapeutic efficacy.
• Magnesium has multiple functions in the treatment of different types of bone defects.
• Review offers new insights for magnesium-based bone implants and Mg^2+^-functionalized bone implants graft design.

## Graphical abstract

**Figure:**
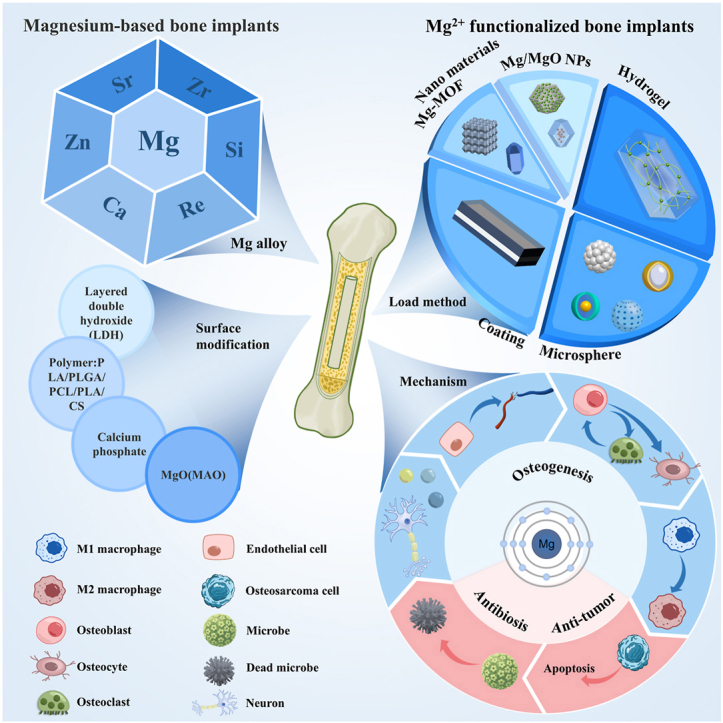

## 1 Introduction

Bone is a dynamic hard tissue composed of living cells and minerals, characterized by its remarkable capacity for self-healing after injury [1]. However, self-healing is challenging in the presence of massive bone defects due to trauma, resection of tumors, and infections. Numerous factors influence the outcome of treatment after a bone injury, including the location and length of the defect, the condition of the soft tissue capsule, and the mechanical environment, among others. Once the size of the bone defect exceeds a critical value, the potential for self-healing becomes markedly limited [2,3]. The treatment of severe bone defects usually requires the implantation of a bone substitute. As the gold standard for bone defects, autologous bone grafting has remarkable efficacy; however, it is limited by the restricted amount of bone in donor areas and donor complications [4,5]. Although homogeneous allogeneic bone is an alternative to autologous bone, its biological activity is low, with a high risk of pathogen infection and immune rejection [5,6]. Therefore, researchers have explored numerous biomaterials as suitable bone substitute [7].

Magnesium is an essential trace element, the fourth most abundant cation in the human body, and is involved in various physiological and metabolic activities [[8], [9], [10]]. As a novel biomaterial, magnesium-based materials not only exhibit biodegradability but also possess an elastic modulus similar to that of human bones, which aids in reducing the stress shielding effect, when compared to traditional bio-metal materials such as titanium alloys, stainless steel, and cobalt-based alloys [11]. Although its degradability avoids the need for secondary surgical removal, its consequent rapid degradation and local hydrogen production limit its use in promoting bone regeneration [12,13]. The most suitable degradation rate should match the process of bone repair. Therefore, improvement strategies of magnesium alloy materials have been extensively studied, and have focused on alloy preparation and surface coating techniques [14,15]. Alloying can improve the degradation performance of magnesium through grain refinement and solid solution strengthening. On the other hand, surface modification strategies, represented by micro-arc oxidation and organic polymers, slow the degradation of magnesium by physically isolating the corrosive environment and are more effective than alloying.

Besides acting as a supporting scaffold to provide a mechanical microenvironment for the bone defect site, Mg also plays a significant role in the bone repair process [16,17]. Mg promotes the differentiation and adhesion of osteoblasts, and it regulates the balance between osteogenesis and osteoclastic activity [18]. Additionally, Mg indirectly regulates bone regeneration by immunomodulation and promotes neurovascular reconstruction [19,20]. Therefore, biomaterials containing Mg have been extensively developed and proven to have excellent osteogenic effects. Currently, research on the application of magnesium-related bone implants in bone defects mainly focuses on the mechanical properties and degradation of magnesium alloys, and the summary of magnesium's bone repair mechanism is relatively limited. Starting from the multiple etiologies of bone defects and the complex internal microenvironment, this review discusses magnesium's role in various dimensions, including direct osteogenesis, inhibition of osteoclasts, immune modulation, neuro-osseointegration, vascularized bone regeneration, antitumor effects, and antibacterial effects.

**Fig. 1:**
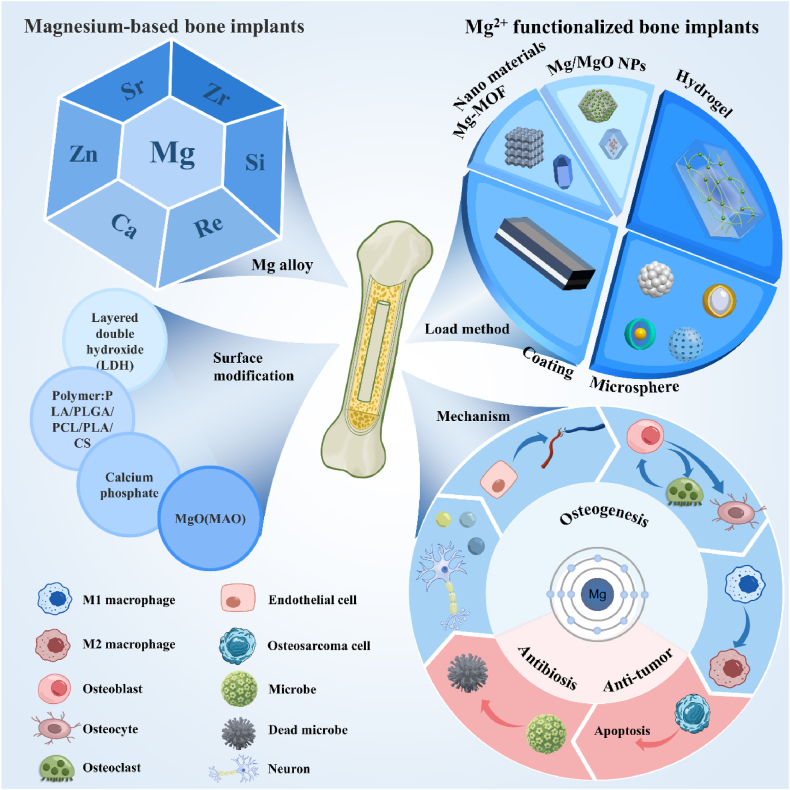
The schematic diagram of modification method, loading method and action mechanism of magnesium in the treatment of bone defects.

In this review, magnesium-based bone implants that employed the mechanical properties and degradation characteristics of magnesium as their cornerstone were analyzed and discussed. The advantages and disadvantages of their application were outlined, as well as ways to improve them. Subsequently, the magnesium-functionalized modified bone implants based on the functionality of Mg^2+^ regulating cell behavior was summarized and discussed. The specific mechanisms of action of magnesium in direct osteogenesis, vascularized osteogenesis, neuralized osteogenesis, antimicrobial, and tumor inhibition were analyzed, and various methods for loading magnesium as a functional constituent were described (Fig. 1). This review provides the theoretical basis and design strategy for the clinical application of magnesium-based materials, and suggests new strategies for the treatment of bone defects.

## 2 Magnesium-based bone implants

Magnesium-based metals are mechanically stronger than polymers and biodegrade better than permanent metals, setting a new standard for temporary implant applications in musculoskeletal surgery [21]. This section discusses the advantages and application ideas of magnesium-based metals as bone support scaffolds for the treatment of bone defects.

### 2.1 Mechanical properties and degradability of magnesium scaffolds

Magnesium offers a new option for bone implants due to its special mechanical properties [22]. The density and elastic modulus of magnesium are similar to those of cortical bone; hence, magnesium-based bone implants effectively avoid stress shielding [23,24]. However, the hardness and compressive strength of magnesium are slightly lower than those of normal bone tissue [21]. Magnesium needs to be modified by alloying or improving the forging process to enhance its mechanical strength. The benchmarks of performance for bone implants include a tensile yield strength >200 MPa, an ultimate tensile strength >300 MPa, and an elongation >10 % [25].

Biodegradability confers a unique advantage to magnesium in the preparation of bone implants, as this makes it unnecessary to remove it after implantation for local bone defects [12]. With the degradation of magnesium, magnesium-based scaffolds gradually provide space for the migration of bone cells and the growth of blood vessels.

Suitable degradable orthopedic implants should provide suitable mechanical properties at the initial stage, and then completely degrade when new bone tissues can provide sufficient local stability, that is, the degradation speed should match the bone repair speed [26,27]. However, current research shows that the degradation of magnesium in the body is rapid, and simple magnesium materials will fail within a short period. High corrosion performance makes magnesium highly sensitive to stress corrosion cracking and corrosion fatigue, which leads to catastrophic fracture of magnesium implants under the synergistic effect of mechanical stress and the corrosive environment created by body fluids, which becomes a major challenge to the use of magnesium and its alloys as bone implants [28]. Chen et al. [29] showed that the mechanical properties of pure magnesium materials under stress degraded to 10–20 % of their original strength within 14 days, and undoubtedly failed to support the early healing of bone defects. However, the mechanical integrity of bone implant materials should be maintained for several weeks (≥24 weeks: the longest healing time of femoral neck fractures) [30].

In the complex body fluid environment, many factors can affect the degradation rate of magnesium, including the concentration of chloride or phosphate ions, pH value, various cytokines, and blood flow rate. Among these factors, the most critical is chloride ion concentration; magnesium is covered by an insoluble layer of magnesium hydroxide during degradation, and chloride ions can destroy magnesium hydroxide as an isolation layer and accelerate magnesium corrosion [31]. Studies have shown that improving the corrosion resistance of magnesium by alloying, optimizing grain size and phase distribution, and isolating the corrosive factors of body fluids by surface treatment are necessary to maintain the mechanical integrity of bone implants [32,33].

The H_2_ produced by magnesium degradation is also a critical factor influencing the successful application of implants. Excessive production and local accumulation of H_2_ lead to the formation of gas cavities in bone defect tissues, resulting in swelling and pain. More seriously, it inhibits physiological activities such as cell migration, adhesion, colonization, and osteogenic differentiation [34,35]. Studies have shown that during the initial bone healing phase, the size of gas cavities is directly proportional to the risk of fixation failure of bone implants [35]. However, the positive effects of H_2_ on osteogenesis cannot be ignored. H_2_ has antioxidant effects, so the hydrogen-rich environment in the body has a protective effect on bone mineral density [36]. Controlled hydrogen levels can rapidly diffuse into surrounding tissues, avoiding the formation of gas cavities and exerting a positive osteogenic effect [37]. The generation rate of H_2_ is also directly proportional to the degradation rate of magnesium; therefore, the key to controlling H_2_ is to control the degradation of magnesium. The release of H_2_ can be limited to a harmless level by improving the corrosion resistance of implants or reducing the ratio of implants to body weight.

Purification, alloying, and surface modification of magnesium are important strategies to improve the performance of magnesium-based stents. High-purity magnesium (HP-Mg) (99.99 wt%) exhibits excellent corrosion resistance, which is attributed to the absence of secondary phases and reduced micro-galvanic reactions [38]. More importantly, high-purity magnesium is a safe implant material because it does not contain toxic alloying elements. However, magnesium purification has limited improvement on mechanical properties, which is not conducive to expanding the application of these materials in weight-bearing areas for fracture fixation [39]. Alloying introduces alloy elements to alter the properties of the alloy through grain refinement, lattice distortion, solid solution strengthening, and secondary strengthening. Additionally, by adjusting the type and content of alloy elements, magnesium alloys possess unlimited possibilities. Surface modification involves the formation of an inert barrier layer on the surface of the magnesium substrate to block corrosive media or introduce corrosion inhibitors to suppress the corrosion process. Some surface strategies even have the capability of self-repairing corrosion pits [40].

### 2.2 Magnesium-based alloy scaffold

Alloying is a highly potential improvement strategy. By adjusting the types and content of alloy elements, the physical, chemical, crystalline, and mechanical properties of the alloy can be altered (Table 1) [32].

**Table 1:** Mechanical properties of magnesium alloys used in bone implants.

Substrate	Condition	YS(MPa)	UTS(MPa)	Elastic modulus (GPa)	Elongation (%)	Ref.
Mg	Purified	148.5	199.1		8.1	[41]
Mg	As cast	27.5	97.5		7.31	[42]
Mg	As extruded		90–105			[41]
Mg-1Ca	As cast	∼135	239.6		10.6	[41]
Mg-2Ca	As cast	47.3	115.2		3.05	[42]
Mg-4Ca	As cast	34.5	77.4		2.10	[42]
Mg-0.5Sr		37	74		2.6	[30]
Mg-2Sr	As cast	∼145	213.3		3.2	[41]
Mg-4Zn	additive manufacturing	14.5		448.8		[43]
Mg-6Zn	As cast	169.5	279.5		18.8	[41]
Mg-2Si	As cast		148			[44]
Mg-4Si	As cast		139			[44]
Mg-6Si	As cast	77	97			[44]
Mg–1Zn–1Mn	As cast	44	174			[45]
Mg–1Zn–1Mn	As extruded	246	280			[45]
Mg-1.0Mn-2Zn	powder metallurgy	232.42				[46]
Mg-1.5Mn-2Zn	powder metallurgy	185.611				[46]
Mg–2Zn–1Mn	Centrifugal cast	259.99				[47]
Mg-1.3Ca-5.5Zr	As cast			26.853		[48]
Mg-xZr-ySr	As cast	65–125	200–290		14–38	[49]
Mg-2Ca-0.5Mn-4Zn	As cast	83.1	189.2		8.71	[42]
Mg-Zn-Zr-Nd	As extruded	256	299	44.61	26.15	[50]
Mg-Zn-Zr-Y	As extruded	276.67	312	45.05	19.89	[50]
Mg-0.6Zr-0.5Sr-2Sc	As cast	77	81		3.2	[30]
Mg-Y-Ca-Zr	As cast	120–150	74–100	34–60		[51]
Mg-3.66Al-4.25Ca-0.43 Mn	hot extrusion		420		7	[52]
Mg-2Ca-1Mn-1 Sr(XMJ211)	permanent mold casting		165.5		8	[53]
AZ31	As extruded		278.29			[54]
AZ91-3Ca			383.2			[55]
WE43		24		0.75		[56]
JDBM	additive manufacturing		97.13	1.98		[14]

#### 2.2.1 Alloying elements used in magnesium-based alloy scaffolds

The properties of magnesium alloys depend on the kind and proportion of their constituent metal elements. The selection of magnesium alloys for bone implants requires careful consideration of their mechanical properties, degradability, biocompatibility, and some osteoinductive activity of the alloying elements. Grain refinement caused by metal element doping can improve the mechanical properties of magnesium, especially with the problem of magnesium's insufficient yield strength. Governed by the Hall-Petch relationship, the room temperature tensile strength of magnesium and its alloys is inversely proportional to the square root of the grain size; therefore, grain refinement is an effective way to improve the strength of magnesium and magnesium alloys [57]. Similarly, alloying obviously inhibits the degradation and hydrogen evolution of magnesium [58]. Electrochemically, a decrease in grain size increases the grain boundary density, which will affect dissolution and passivation. Grain refinement treatment tends to allow more pseudo-protective oxide layers on the surface of magnesium implants [57]. Moreover, alloying can delay cathodic kinetics by poisoning the hydrogen evolution reaction, minimize its influence on the formation of intermetallic compound particle types, and reduce the addition of anodic kinetic solid solution [59]. Common alloying elements involved in magnesium-based implants include calcium (Ca), zinc (Zn), strontium (Sr), rare earth (RE) metals, zirconium, aluminum, and manganese. Table 2 summarizes the effects of these alloying elements on the properties of magnesium alloys.

**Table 2:** Effects of common alloying elements in magnesium-based bone implants on corrosion behavior, biocompatibility, and mechanical properties of the implants.

alloying element	Corrosion Behavior	Mechanical Properties	Biocompatibility
Ca	When the mass fraction is less than 2 %, the corrosion resistance will be improved [42]	Improve strength [60]	Basic elements of the human body; Promote bone healing [60]
Zn	When the mass fraction is between 1 % and 5 %, the corrosion resistance is improved [61,62]	The tensile strength and hardness are enhanced, which first increases and then decreases with the Zn content [63,64]	Essential trace elements in human body [65]
Sr	When the mass fraction is less than 2, the corrosion resistance is improved [66]	When the mass fraction is less than 2, the mechanical strength is improved [66]	Promote the maturation of osteoblasts; Conducive to bone formation [67]
RE	Improve corrosion resistance [68]	Improve mechanical strength and ductility [69]	Bioinert, some RE elements are cytotoxic [70].
Al	The corrosion rate decreases with the increase of Al content [71]	Improve yield strength and ductility [71]	Neurotoxicity [71]
Li	Reduce corrosion resistance [72]	Reduce strength; Improve ductility [73]	Abnormal thyroid function; Renal function injury [74]
Mn	Small addition can improve corrosion resistance [46]	Improve strength and ductility [46]	Essential trace elements; It plays an important role in the immune system; At higher concentrations, it is neurotoxic [46]

Ca is important as an essential mineral element in the composition of human bone tissue. Ca improves the microstructure of implants through grain refinement and the formation of stable intermetallic compounds to improve the mechanical properties and corrosion resistance of magnesium alloys [60,75]. Compared with pure magnesium, the Mg-Ca alloy has increased tensile strength, yield strength, and hardness; moreover, the hardness of this alloy is proportional to the Ca ratio. The tensile strength and yield strength in Mg-2Ca (Mg-2wt%Ca) are the most suitable, which represents an increase from 97.5 MPa to 27.5 MPa for pure magnesium to 115.2 MPa and 47.3 MPa, respectively. On the other hand, with an increase of Ca ratio, the tensile strength and yield strength of Mg-4Ca are reduced to 77.4 MPa and 34.5 MPa, respectively [42]. Furthermore, Ca improved the corrosion potential of Mg-Ca alloy and the passivation of the alloy surface caused by the degradation process, in which the corrosion rate of Mg-2Ca magnesium alloy reached the minimum [76,77].

Zn is an essential micronutrient that, in trace amounts, promotes growth and development. The hardness of the Mg-Zn alloy increases as its Zn content increases; however, the maximum compressive stress and maximum flexural strength initially increase and then decrease as the Zn content increases [65]. This is because micropores are formed due to the different diffusion rates of magnesium and zinc during the synthesis of the Mg-Zn alloy. Furthermore, a high zinc ratio will increase the formation of micro-pores in the alloy, which negatively affects the mechanical strength and offsets the positive effects of grain refinement. The improvement of the degradation rate of the Mg-Zn alloy is also greatly related to the concentration of doped Zn. The concentration of 1 wt% to 5 wt% can improve the corrosion resistance of Mg-Zn alloys, while a concentration above 7 wt% will lead to the corrosion of Mg-Zn alloys because of the network structure of the Mg-Zn intermetallic compound and the accelerated micro-coupling corrosion [61,62]. Zn is not an optimal alloying element for improving the corrosion resistance of magnesium alloys; however, it strengthens the mechanical properties of magnesium alloys and has excellent bioactivity [63,64].

The addition of Sr can effectively improve the mechanical properties and corrosion resistance of magnesium alloys. When the strontium content is 0∼2 wt%, the grain size increases as the strontium proportion increases; this is followed by improvements in mechanical strength and corrosion resistance. When the proportion of Sr is between 3 wt% and 4 wt%, there is no obvious decrease in grain size, but the volume fraction of the Mg_17_Sr_2_ phase increases as the crack source increases, leading to a decrease in mechanical strength [66,78]. However, the corrosion rate of Mg-Sr alloy first decreases and then increases as the strontium proportion increases, and the best corrosion resistance is obtained at 2 wt% [79]. Therefore, it is considered that Mg-Sr alloy with 2 wt% Sr content is the most suitable for bone implants. In addition to improving mechanical properties, strontium also has some biological functions. As a bone-seeking element, it promotes bone regeneration and inhibits bone absorption [67].

Alloying RE elements with magnesium is the main strategy for improving its mechanical properties. RE elements known to have research applications in magnesium alloys include cobalt (Co), gadolinium (Gd) [80], neodymium (Nd), lanthanum (La) [69], etc. RE elements strengthen the mechanical strength and corrosion resistance of magnesium alloys mainly through grain refinement and weaken the texture of magnesium alloy, thus improving the ductility of magnesium alloys [68]. The modification emphases of different RE vary, and the addition of Co and La is better for improving the strength of the alloy. Microalloying with Nd and Gd leads to an increase in elongation by 1.5–2 times. Compared with magnesium alloys that do not contain RE, the addition of RE decreases the corrosion rate by 45–66 % [81]. However, the biocompatibility of RE elements is their main disadvantage. Some rare earth elements, such as cerium and praseodymium, have significant cytotoxicity and need to be carefully introduced [70].

#### 2.2.2 Application of magnesium-based alloy scaffolds for bone repair

According to the number of alloying elements, magnesium alloys can be categorized into binary magnesium alloys, ternary magnesium alloys, quaternary magnesium alloys, and others. Binary magnesium alloys have been widely studied; however, the improvement of mechanical properties, corrosion resistance, and biological activity of magnesium alloys obtained by adding a single element is limited. Therefore, ternary, quaternary, and other multicomponent alloys have been developed, further improving the properties of alloys [82,83].

Currently, commonly used multi-element alloys, such as commercial AZ31 and WE43 magnesium alloys, have been widely studied and applied, and their mechanical properties, ductility, and corrosion resistance have been reliably verified [[84], [85], [86]]. AZ31 uses Al, Si, Ca, Zn, and other trace elements as alloying elements. AZ31 is easy to obtain and has excellent processability. It is a good magnesium substrate that is suitable for various surface modifications, making it one of the most studied magnesium alloys for bone implants [87]. The WE series alloy is an Mg-Re alloy to which various Re elements have been added. Its representative alloy is WE43, an alloy with 43 wt% of Y added [84]. In recent years, several researchers have explored the application of WE43 alloys in bone implants, conducted various surface modification studies for them, and concluded that WE43 alloys are an important option for magnesium-based bone implants [[88], [89], [90]]. AZ31 and WE43 alloys have been successfully used in bone repair; however, the element composition and proportion of commercial magnesium alloys are not completely in line with the microenvironment of the human body. Researchers are also exploring the composition and proportion of magnesium alloys that are more suitable for the treatment of bone defects.

The recent application of ternary magnesium alloys, represented by the Mg-Zn-Ca alloy, in bone implants has been widely studied, and both Ca and Zn have been identified as excellent choices for doping magnesium alloys. Compared with binary magnesium alloys Mg-Ca and Mg-Zn, ternary magnesium alloys Mg-Ca-Zn have better mechanical properties and corrosion resistance [76,91]. The intermetallic compound Ca_2_Mg_6_Zn_3_ formed in the Mg-Zn-Ca alloy is responsible for enhancing the ductility of the alloy [79]. Moreover, Zn^2+^ and Ca^2+^ are both bioactive factors that induce osteogenesis; additionally, their synergistic osteogenesis effect is better. Therefore, the improvement of the corrosion resistance of the alloy by Zn and Ca can be achieved within a certain concentration range. When Zn exceeds 7 wt% and Ca exceeds 2 wt%, the corrosion resistance of the alloy will be adversely affected. Micro-doping of Zn and Ca is a suitable method for improving the corrosion resistance of Mg-Zn-Ca [61,77]. In a study of bone implants based on Mg-Zn-Ca alloys, the research team explored the different doping ratios of Ca and Zn to obtain the mechanical properties and corrosion resistance that best meet the requirements of bone implants. Mao et al. [92] developed an Mg-2Zn-0.05Ca alloy with balanced mechanical properties and corrosion resistance, which is expected to improve the application prospects of bone implants.

Similarly, the performance improvement of quaternary magnesium alloys comes from the further grain refinement and the formation of various intermetallic phases caused by the addition of more elements. Mg-Nd-Zn-Zr (JDBM) is a representative quaternary magnesium alloy used in bone implants that has matching strength, uniform degradation mode, and good biocompatibility [93]. JDBM creates a reasonable Mg^2+^ microenvironment by virtue of its uniform degradation, as it shows outstanding osteogenic activity [94]. JDBM has been applied to the treatment of medial malleolus fractures and achieves good fracture reduction and functional recovery. Among them, JDBM gradually degenerates within 12 months after implantation, and the implant does not break apart before fracture healing, which proves that it has a good clinical transformation prospect in bone repair [95].

### 2.3 Surface-modified magnesium-based scaffolds

In addition to alloying, surface modification effectively enhances the corrosion resistance of magnesium and reduces its degradation rate [96]. Through surface modification, an inert barrier is formed on the surface of the magnesium substrate to block the corrosion medium or introduce a corrosion inhibitor to inhibit the corrosion process. Some modification strategies also enable the self-repair of eroded pits [40]. Surface modifications, including oxide coatings, layered double hydroxides (LDH), inorganic coatings, and organic polymer coatings, will be further discussed in this section. Table 3 summarizes the corrosion test results of various magnesium alloys and surface modifications.

**Table 3:** Corrosion properties of magnesium alloys and surface modifications.

Substrate	Coating	Technology	Solution	Corrosion studies				Ref.
				E_corr_ (V_SCE_)	I_corr_ (μA·cm^−2^)	β_c_ (mV/dec)	Corrosion rate (mm·y^−1^)	
Mg			SBF	−1.942	298.4			[97]
Mg			Hank's	−1.55	7.8			[98]
Mg	MgO	Micro-arc oxidation (MAO)	SBF	−1.888	60.48			[97]
Mg	Phytic acid &zoledronic acid	Dip-coating	SBF	−1.91	36.96			[99]
Mg-Zn-Ca			Hank's	−1.74	86.70	36.4	0.7	[100]
Mg-Zn-Ca	NaMgF3	Dip-coating	Hank's	−1.45	6.49	28.1	0.4	[100]
Mg-3Zn-0.2Ca			SBF	−1.461	101.25			[101]
Mg-3Zn-0.2Ca	Ag/ZIF-8/LDH	Hydrothermal deposition	SBF	−1.171	0.43			[101]
Mg-2Zn-0.2Ca			Hank's	−1.388	0.4132			[91]
Mg-2Zn-0.2Ca	CaCO3/DOPA		Hank's	−1.390	0.01165			[91]
Mg-1Zr-1Ca			SBF		0.43			[102]
Mg-1Zr-2Sr			SBF		499.62		14.39	[103]
Mg-6Sn-5Zn			Hank's	−1.57	14		0.32	[98]
Mg-Ca-Zn-Ag			FBS	−1.48	58.9	−85.61		[104]
Mg-Ca-Zn-Ag	hydroxide/graphene oxide/hydroxyapatite	Electrochemical deposition;	FBS	−0.41	0.912	−187.23		[104]
AZ31			PBS	−1.56	19.8			[105]
AZ31	MgO	MAO	PBS	−1.53	0.0436			[105]
AZ31			SBF	−1.60	231	376	0.935	[86,106]
AZ31(H+)			SBF	−1.56	103			[86]
AZ31	HA	Hydrothermal deposition	SBF	−1.26	6.10			[107]
AZ31	GCC	Dip-coating	SBF	−0.541	0.266	75	0.006	[106]
AZ31	LDH	Hydrothermal deposition	PBS	−1.56	1.42			[108]
AZ31	PCL	Electrospinning fabrication	PBS	−1.52	63			[109]
AZ31	BG	Sol-gel treatment	SBF	−1.759	381.81			[40]
AZ31	LDH	In-situ hydrothermal growth	SBF	−0.255	3.13			[40]
AZ31B			SBF	−1.758	65.93		1.49	[110]
AZ31B	CMP	Sol-gel treatment	SBF	−0.626	0.03775			[111]
AZ60			SBF	−1.43	67			[112]
AZ60	CaP	Dip-coating	SBF	−1.35	6			[112]
AZ91			SBF	−1.42	34.2	272.7	29.9	[113]
AZ91	chitosan/ZIF-8 MOF	Dip-coating	SBF	−1.45	6.5	169.1	5.6	[113]
AZ91D	ZrO_2_	Plasma spraying	3.5 wt % NaCl solution	−1.12	0.467			[114]
WE43			SBF	−1.87	286	−0.36		[88]
WE43	Ca-Zn-P/Zn		SBF	−0.97	0.034	−0.31		[88]
ZK60			SBF	−1.649	10.3			[115]
ZK60	HA	Plasma spraying	SBF	−1.490	1.169			[115]
ZK60	Nb/ZrO_2_/HA	Plasma spraying	SBF	−1.364	0.51			[115]
JDBM			SBF				0.039	[14]

#### 2.3.1 Oxide-coated magnesium-based scaffolds

The advantages of oxidative modification on magnesium-based scaffolds include improved corrosion resistance, wear resistance, biocompatibility, and interfacial adhesion [97]. Common techniques for surface modification of magnesium-based scaffolds with complex geometric shapes include Micro-arc Oxidation (MAO) and High-Temperature Oxidation (HTO) [116,117]. The principle lies in the shaping of oxides formed by the chemical transformation of magnesium substrates on magnesium-based scaffolds by means of a micro-arc oxidation process and the outward and inward growth of the oxides [105]. The advantages of MAO modification on magnesium-based implants are improved corrosion resistance, wear resistance, biocompatibility, and interfacial adhesion [97]. MAO has an obvious effect on improving the corrosion resistance of magnesium-based scaffolds, which is reflected in the obvious decrease in corrosion current density (Icorr) of the whole material after MAO treatment. The electrochemical test results of Zhang et al. show that the Icorr of AZ31 alloy is 19.8 μA cm^−2^; furthermore, the Icorr of AZ31 alloy after MAO treatment is reduced to 0.0436 μA cm^−2^ (Table 2), and the corrosion performance is greatly improved, so as to enable magnesium-based bone implants to maintain sufficient support time in the bone defect [105]. MAO-modified coating is porous, which improves its biocompatibility and makes it very suitable for cell adhesion and proliferation [118,119]. This porosity may be caused by the formation of more pronounced macroscopic pores near the surface of the MAO coating, which may allow for the precipitation of oxygen. The important role of MAO modification is also that it can provide a basis for further construction of composite coatings with even better properties. Li et al. [120] developed a new bisphosphonate (BP)-loaded microarc oxidation (MAO) coating by chemically conjugating nitrogen-containing BP with the MAO coating and then piggybacking the BP on the MAO coating. The BP release exerts an inhibitory effect on osteosarcoma, which provides a new vision for the application of magnesium-based bone implants for bone defects after tumor resection. HTO involves reacting the surface of magnesium-based scaffolds with oxygen at high temperatures to form oxides. Besides oxide formation, HTO treatment can induce element migration and layered structures, resulting in a better isolation layer structure [121]. Liu et al. [117] demonstrated that magnesium-based scaffolds treated with HTO exhibit adequate structural and mechanical properties, with a significantly reduced degradation rate, and do not cause cytotoxicity or biorejection.

#### 2.3.2 Calcium phosphate-coated magnesium-based scaffolds

Calcium phosphate represents a significant example of bioceramic materials applied in bone implants. While bioceramics exhibit excellent chemical stability and bioactivity, their inherent brittleness and limited mechanical properties often restrict their use in weight-bearing applications. In contrast, magnesium, with its suitable Young's modulus and superior ductility, serves as an ideal supporting material. Bioceramics, on the other hand, constitute an excellent coating choice for magnesium-based bone implants [55,64]. There are various calcium phosphate compounds, such as calcium phosphate, calcium metaphosphate, dicalcium phosphate dihydrate, and HA, Calcium phosphate coatings are an attractive surface modification strategy for magnesium-based scaffolds to improve their corrosion resistance while endowing them with bone conduction performance. Furthermore, simple calcium phosphate coating modification can be used as a physical barrier to slow down degradation and reduce hydrogen release; however, it can enhance surface hydrophilicity and enhance cell adhesion [122]. The results of electrochemical tests and immersion experiments show that the corrosion current density of magnesium substrate modified by calcium phosphate significantly reduces, and the degradation rate slows down significantly within 14 days. Furthermore, calcium phosphate coating promotes the proliferation, adhesion, and diffusion migration of bone marrow stem cells on implants [23]. Moreover, some functional metal elements doped with calcium phosphate coatings can confer magnesium-based scaffolds with more functions, such as strontium-doped calcium phosphate coatings with stronger osteogenic properties [123]. Additionally, calcium phosphate can be mixed with other materials, such as chitosan, graphene, and magnesium hydroxide, among others, to obtain a mixed coating to modify the surface of magnesium-based materials. Furthermore, the mixed coating has better corrosion resistance and better biocompatibility [104]. Moreover, just as the addition of graphene improves the corrosion resistance of mixed coatings, the addition of chitosan confers the coating with drug-loading characteristics [107,124].

#### 2.3.3 Polymer-coated magnesium-based scaffolds

Organic polymers exhibit good plasticity and biodegradability; however, their low mechanical strength limits their application in load-bearing sites. Combining them as coatings for magnesium-based materials, which possess excellent comprehensive mechanical properties, presents an excellent complementary strategy [125]. Two kinds of polymers are commonly used for surface modification of magnesium-based bone implants, namely, natural polymers such as chitosan and collagen, and other synthetic polymers such as polyethylene glycol, polylactic acid, and polycaprolactone [126].

The good plasticity of the polymer coating makes it easy to form a closed isolation layer that effectively improve corrosion resistance. Furthermore, it is considered to have the ability to control and guide life activities, such as cell proliferation, adhesion, and differentiation. Moreover, the antibacterial properties and encapsulation properties of organic polymer materials make them an excellent choice for drug-loaded coatings and antibacterial coatings on magnesium-based bone implants. The plasticity and biodegradability of organic polymers make it an excellent choice for the preparation of hydrogels and microspheres for the carriage and release of drugs. The antibacterial mechanisms of polymers lie in their electrostatic interactions and antibacterial groups [127]. The positively charged groups in the polymer, such as amino groups and quaternary ammonium groups, can interact with negatively charged microbial biofilm to achieve antimicrobial effects [128]. Zhang et al. [129] prepared chitosan/poly-L-glutamic acid coating on AZ31 alloy using a lamination method, and proved its successful result in improving corrosion resistance and antibacterial properties of magnesium substrate.

#### 2.3.4 Layered double hydroxide-coated magnesium-based scaffolds

LDHs are a class of anionic clays, consisting of metal ions and anions, usually represented by a sandwich structure. It is a 2D material with high plasticity and good biocompatibility, making it an excellent choice for magnesium-based scaffolds coating [130,131]. The coating methods of LDH on magnesium-based scaffolds include the in-situ growth method, hydrothermal deposition method, electrochemical deposition method, and anion exchange method. Through chemical bonding, LDH and magnesium substrate can be stably attached [131].

LDH has a unique effect on improving the corrosion resistance of magnesium-based scaffolds because it not only acts as a physical barrier but can also absorb corrosive anions, such as Cl^−^ in body fluids due to its anion exchange characteristics [40,131]. Moreover, LDH itself can be used as an ion pool, and its layered structure confers it with drug loading properties, which can further enhance the corrosion resistance of magnesium matrix by loading corrosion inhibitors or drugs to exert corresponding osteogenic, antibacterial, and antitumor activity. LDH has photothermal properties, which enable it to modify the antibacterial and antitumor properties of magnesium-based scaffolds. Cheng et al. [132] constructed LDH-Mn coating loaded with celastrol on the surface of magnesium alloy, which effectively kills osteosarcoma in vivo and in vitro through photothermal therapy and the release of celastrol. Similarly, it stably releases Mg^2+^ and Mn^2+^, showing good biocompatibility and osteogenic activity.

## 3 Mg-functionalized bone implants for bone defects

### 3.1 Mg nanoparticles

**Fig. 2:**
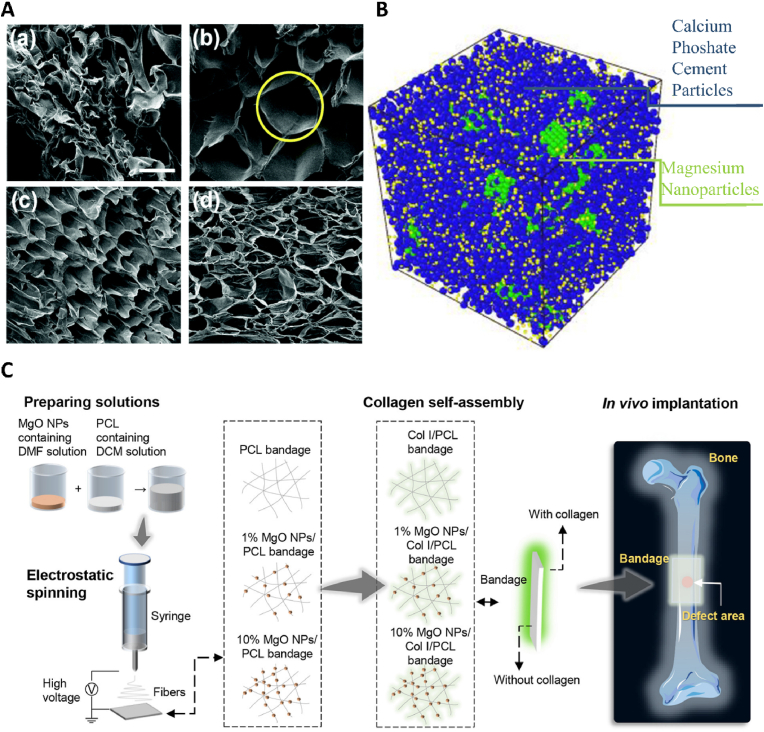
(A) SEM images of the cross section of lyophilized hydrogels. (a) 0NH, (b) 0.3 MH, (c) 0.5 MH, (d) 0.7M [140]. (B) A view of the reinforced cement atomic structure by magnesium nanoparticles [145]. (C) MgO NPs-carried artificial periosteal bandage was prepared by electrospinning MgO NPs and PCL Collagen was self-assembled onto the surface of periosteal bandage in situ before implantation in vivo [146].

Nanomaterials have unique physical and chemical properties and microstructures, especially the tiny size of nanomaterials makes them have a larger surface area and higher reactivity. Nanostructures with cellular-friendly surface features can promote higher-order protein interaction [133,134]. Magnesium nanoparticles can be used as a Mg^2+^ reservoir, crosslinker, antibacterial substance, photothermal agent, and drug loading platform [135]. Mg-NPs and MgO-NPs are widely used magnesium-related nanoparticles. As excellent Mg^2+^ reservoirs, their osteogenic properties have been widely verified. Among them, MgO reduces the degradation rate to a certain extent compared with Mg, and avoids the generation of hydrogen [136]. Mg/MgO-NPs are often incorporated into matrix materials such as electrospun fibers, hydrogels, and bone cements for application in bone implants, as their independent use faces challenges like poor mechanical strength and rapid degradation [137] (Fig. 2). Mg/MgO-NPs can form chemical bonds or undergo physical adsorption with matrix materials, thus improving the structure and mechanical properties of hydrogel, bone cement, and other materials [135]. Just as Mg-NPs and calcium phosphate cement have proved to be good partners, the addition of Mg-NPs enhances the strength of calcium phosphate cement and regulates its porosity [138]. The method of loading MgO-NPs on hydrogel depends on the adsorption characteristics of nanomaterials, and the unique crosslinking agent and stabilizer properties of MgO-NPs. Because Mg(OH)_2_ forms on the particle surface when MgO-NPs meets water, it can chelate with acidic groups in hydrogel [9,139]. Pan et al. [140] added MgO/NPs to gelatin methacryloyl (GelMA)-co- polyethylene glycol (PEG) hydrogel through its crosslinking agent characteristics. As an emulsion stabilizer, MgO/NPs improved the mechanical properties of GelMA-co-PEG hydrogel and formed a specific macroporous structure (Fig. 2A), which effectively supported the in-situ bone regeneration of rat bone defects. Apart from its osteogenic activity, Mg/MgO-NPs is also an important antibacterial material. Moreover, it has been successfully used as a photothermal agent in antibacterial and anti-tumor treatments. Additionally, MgO-NPs can be used as a potential platform for loading metal ions because of its high surface activity and good adsorption capacity for heavy metal ions. Some researchers have used the adsorption characteristics of MgO-NPs to construct a more effective antibacterial nanoparticle, and accurately loaded Ag with MgO-NPs to realize the stable release of Ag [141]. The unique advantage of nanoparticles in exerting their effects in vivo lies in their ability to internalize and function effectively. Nanoparticles enter cells through endocytosis and reach their target sites, thereby exerting their biological functions precisely and efficiently [142]. The internalization efficiency of nanoparticles is influenced by their physicochemical properties, including size, shape, lipid solubility, surface charge, and more [143]. For instance, Lu et al. designed magnesium silicate nanoparticles whose internalization mechanism relies on the positive charge on the nanoparticle surface, which facilitates binding to the negative charge on the cell membrane, enabling their absorption by osteoblasts [144].

Metal organic framework (MOF) is a special kind of nanoparticle, which is a specific framework structure formed by metal ions and organic ligands. In terms of structure and texture, MOF has the characteristics of high porosity, a large surface area, and an adjustable structure, which is the spatial basis of its drug loading [147]. Regarding biological activity, MOF is an excellent repository of ions and organic components, and has both the dual activities of metal ions and organic ligands [148]. Therefore, Mg-MOF has attracted researchers' attention as a Mg^2+^ reservoir and drug delivery system. And magnesium has high solubility due to its group II element characteristics, which endow Mg-MOF with relatively rapid pharmacokinetics [149]. Rarely, compared with other organic chemical materials, Mg-MOF has low cytotoxicity, which makes it a promising bone implant material.

**Fig. 3:**
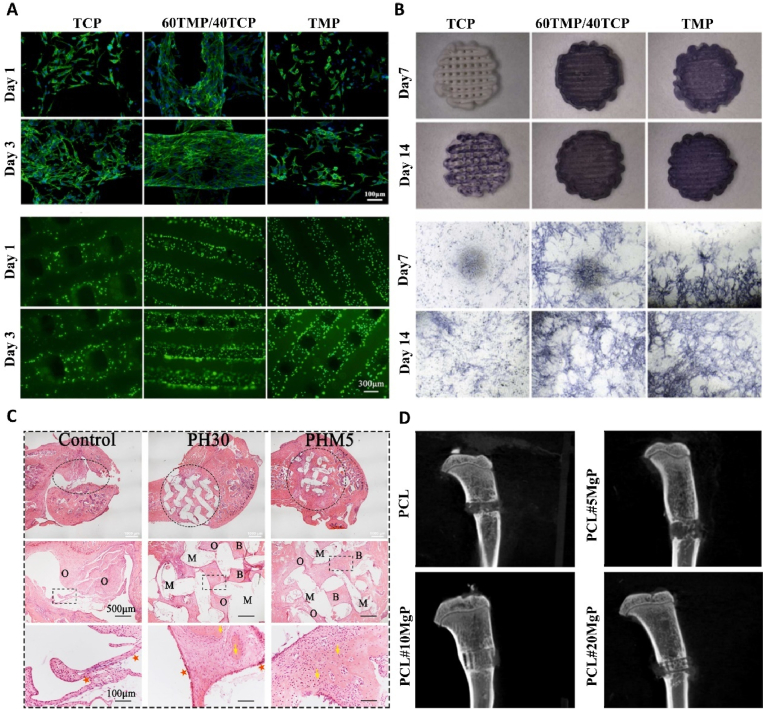
(A) Confocal photographs of cytoskeletons of mBMSCs attaching on the scaffolds [158]. (B) Fluorescent images of viable and dead cells on the scaffolds [158]. (C) H&E staining of bone formation for 4 weeks [159]. (D) X-ray images of tibial defect areas in rats implanted with scaffolds after 8 weeks [160].

At present, the organic ligands of Mg-MOF that are widely used in bone implants are 2,5-dihydroxyterephthalic acid and gallic acid [150]. These two kinds of Mg-MOF have been successfully applied in the treatment of bone defects, and their dual properties as Mg^2+^ carriers and drug-carrying platforms make them beneficial in building drug delivery systems that combine the synergistic effects of multiple drugs. In line with this, Wang et al. [151] loaded icariin with Mg-MOF-74 as the carrier, and proved the stable adsorption of icariin by Mg-MOF-74 and the gradient release of Mg^2+^ and icariin, thus constructing an excellent drug delivery system (Fig. 3A and B). The drug delivery system showed good biocompatibility (Fig. 3C), and the synergistic osteogenesis of Mg^2+^ and icariin was particularly prominent (Fig. 3D–G). Although Mg-MOF has a more stable structure than Zn-MOF, Ni-MOF, and Co-MOF, it still lacks stability in body fluids as a bone implant material [152]. Combining with other materials to reduce the contact between the magnesium MOF and the environment is one of the solutions. Silk fibroin is used to construct cells and extracellular matrix(ECM) structure to protect Mg-MOF [151]. Additionally, researchers designed nanoscale mesoporous silica and calcium phosphate cladding to encapsulate Mg-MOF, thus slowing its degradation rate [153,154].

### 3.2 Mg-incorporated 3D-printed scaffolds

The introduction of Mg-incorporated 3D-printed scaffolds represents a novel approach in bone tissue engineering. By harnessing 3D printing technology, stable combinations of magnesium and other materials can be achieved, addressing the demand for highly complex and precise implant structures in bone tissue engineering. These scaffolds, which integrate the osteoinductive activity of magnesium with the mechanical properties and biodegradability of the base material, emerge as a promising option for bone implants [155]. Researchers in the field of bone tissue engineering tend to design materials that mimic natural bone, aiming to replicate normal bone structure to the greatest extent possible. Natural bone tissue is a heterogeneous material composed of inorganic minerals and organic collagen proteins, and 3D printing technology offers the potential to fabricate biomimetic bone implants with complex structures [156]. 3D printing technology enables precise control over the microstructure of implants, allowing them to possess porosity and pore size distribution akin to natural bone. These porous structures facilitate cell adhesion, proliferation, and differentiation, promoting neovascularization and thereby enhancing osseointegration between the implant and surrounding bone tissue [157]. Commonly used base materials primarily consist of organic polymers and inorganic bioceramics.

Bioceramics, owing to their elemental similarity to natural bone and suitable mechanical properties, exhibit potential in bone implant applications. The incorporation of magnesium further enhances the osteoinductive activity of these bioceramics. 3D printing technology facilitates the seamless integration of magnesium into bioceramics, overcoming challenges associated with conventional manufacturing processes of highly hard and brittle bioceramics, such as high wear rates, structural limitations, and lower precision [155]. He et al. [158] have demonstrated the superior compressive strength and the ability to promote osteoblast proliferation, osteogenic differentiation, and bone-related gene expression of 3D printed Ca_3_Mg_3_(PO_4_)_4_-based bioceramic scaffolds through pneumatic extrusion (Fig. 3A and B).

Organic polymers are widely used in 3D-printed bone implant scaffolds due to their excellent biocompatibility, biodegradability, and ductility. The incorporation of magnesium and magnesium oxide can regulate the degradation of organic polymer scaffolds by inhibiting their autocatalytic reaction, thus preventing catastrophic collapse [159]. Additionally, the OH^−^ formed during magnesium degradation can neutralize the acidic microenvironment generated by the degradation of organic polymers, exhibiting significant osteogenic capability after implantation in vivo [159] (Fig. 3C). Lei et al. [160] prepared a magnesium phosphate-poly (ε-caprolactone) (MgP-PCL) composite scaffold using fused deposition modeling. Their results demonstrated that the incorporation of magnesium phosphate enhances the compressive strength of the polycaprolactone scaffold. Furthermore, MgP-PCL scaffolds demonstrated excellent bone repair effects in vivo experiments (Fig. 3D).

### 3.3 Mg-loaded microsphere

**Fig. 4:**
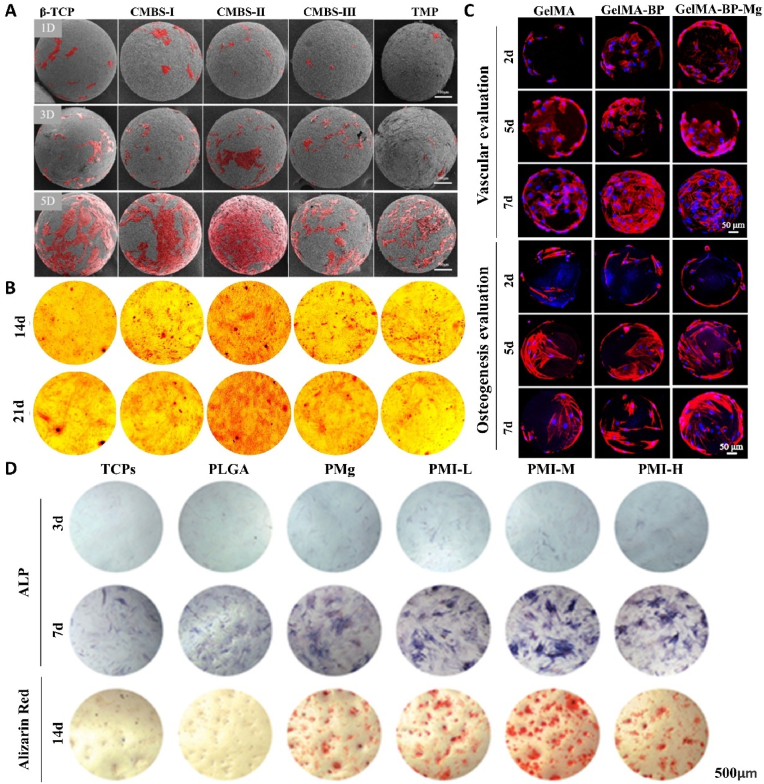
(A) Cell morphology observed under SEM [164]. (B) Alizarin Reds staining at 14 and 21 days [164]. (C) Proliferation behavior of BMSCs and HUVECs cells on the composite microspheres [169]. (D) ALP staining and Alizarin red staining images of BMSCs cultured on different substrates to illustrate the extents of their osteogenic differentiation [170]. (For interpretation of the references to colour in this figure legend, the reader is referred to the Web version of this article.)

Microsphere is a drug delivery method that is of great significance in drug release kinetics and drug targeted delivery [161]. Many studies have used porous microspheres loaded with Mg^2+^ to treat bone defects. Mg^2+^ needs to exert its osteogenic effects in a specific concentration range, and microspheres can carry large doses of drugs and accurately control drug release [162]. Magnesium-based microspheres feature a porous structure that favors cell adhesion, with magnesium evenly distributed within, enhancing the contact area and frequency of Mg^2+^ with cells. This allows for a more effective utilization of the positive effects of Mg^2+^ on osteoblasts [163,164] (Fig. 4).

The design concept of magnesium-based microspheres is extremely rich. The simple synthesis of microspheres is to physically mix magnesium and matrix materials in proportion, so the physical and chemical properties of the synthesized microspheres depend on the matrix materials as a whole [165]. Commonly used microsphere matrix materials include poly(lactic-co-glycolic acid), chitosan, and gelatin, among others. Complex microspheres often involve chemical reactions between magnesium and matrix materials, which can improve the physical and chemical properties of the microsphere matrix and realize a more stable loading of magnesium. The chemical substitution ability of magnesium plays a key role in the synthesis and degradation of magnesium-based microspheres. The most representative example of this ability during synthesis is magnesium's ability to replace calcium in hydroxylapatite(HA) to form Mg-HA microspheres, while optimizing its mechanical strength and increasing its solubility [166]. In the process of degradation, Mg^2+^ can replace the ion bridge structure formed by matrix materials, such as alginate and Ca^2+^, to form diffusion bonds, which regulate the degradation of microspheres and the release of Mg^2+^ [167]. Additionally, Mg^2+^ synthesize microspheres by participating in biomineralization. For instance, Cai et al. [168] synthesize silk fibroin methacryloyl (SilMA) microspheres by microfluidic control, and embedded MgP in SilMA microspheres by in-situ enzymatic reaction, which made its surface and internal structure undergo in-situ biomineralization, thus realizing the modification of the structural properties of the microspheres and the stable loading and sustained release of Mg^2+^. Mg^2+^ can form chemical bonds with some cation-affinity microsphere materials, which is beneficial to the formation of stable microsphere structures and gives microspheres the ability to collect and attract Mg^2+^. Zhao team [169] designed GelMA-BP-Mg microspheres that can dynamically capture Mg^2+^ through the excellent binding affinity between BP and cations. GelMA-BP-Mg microspheres designed in this way capture 0.6 % of Mg^2+^, and the captured Mg^2+^ is effectively released within 18 day, activating osteoblasts and vascular endothelial cells in local bone defect areas, and inhibiting osteoclasts, thus achieving favorable osteogenesis and angiogenesis effects.

### 3.4 Mg-incorporated hydrogel

Hydrogels have become an effective strategy to carry metal ions and drugs because of their plasticity and self-healing [171]. The relatively loose pore structure of hydrogel determines its excellent drug controlled release function. Furthermore, its bionic structure, which is similar to that of the ECM, confers it with excellent biocompatibility, which is beneficial to the adhesion and proliferation of cells on it. Hydrogel loaded with Mg^2+^ has achieved excellent results in the treatment of bone defects.

The loading strategy of hydrogel can be divided into two modes: physical crosslinking and chemical crosslinking [172]. The bonds formed by physical crosslinking are not stable and reversible; hence, the mechanical properties of hydrogels are poor, but they retain good medium fluidity and flexibility. Therefore, it is often used for the treatment of stress-free bone defects or in conjunction with hard materials [173]. The chemical bonds formed by chemical crosslinking are relatively stable, which enhances the mechanical properties of hydrogel and the stability of drug loading [174]. Mg^2+^ itself can serve as a cross-linker, forming connections with groups on the hydrogel matrix material or with introduced intermediate cross-linkers. This enhances the mechanical strength of the hydrogel, stabilizes its shape, and ensures a more uniform pore distribution. Consequently, this imparts Mg^2+^ with more stable release kinetics [175]. Furthermore, Mg^2+^ can form dynamic and reversible coordination bonds with certain groups of the matrix material, imparting better plasticity to the hydrogel. Through the dynamic dissociation and reorganization of its cross-linked structure, the hydrogel can effectively transfer and reshape itself to adapt to the mechanical changes of bone defects [176]. Chen et al. [171] prepared hydrogels loaded with Mg^2+^ based on quaternized chitosan (QCS), and proved that Mg^2+^ combined with QCS through metal coordination to maintain the stability of the hydrogel network. Some studies have chosen to introduce intermediate crosslinking agents such as BP, proanthocyanidins (PC) and PEG to crosslink hydrogels and Mg^2+^ [171]. Li et al. prepared the metal phenol complex Mg-PC of Mg^2+^ and PC through the reaction between metal ions and phenolic molecules, and at the same time, the gel was realized by hydrogen bonding between the phenolic groups of PC and amino hydroxyl carboxyl groups in the polymer [177,178]. As a bridge between hydrogel and Mg^2+^, PC helps to realize the stable loading and slow release of Mg^2+^. The hydrogel has uniform and reasonable pore, and continuously and effectively releases Mg^2+^ and proanthocyanidins, showing excellent osteogenic effects during in vitro and in vivo experiments. Physical crosslinking and chemical crosslinking have their own advantages and disadvantages, and researchers believe that sequential physical and chemical crosslinking hydrogels can be designed by combining their advantages. A study have combined the physical crosslinking between Mg^2+^ and SO_3_^2−^ with the chemical crosslinking formed by the polymerization of methacrylic acid groups in the base polymer chain under ultraviolet irradiation, and invented injectable hydrogels with stable mechanical strength [172].

### 3.5 Mg-containing coating

**Fig. 5:**
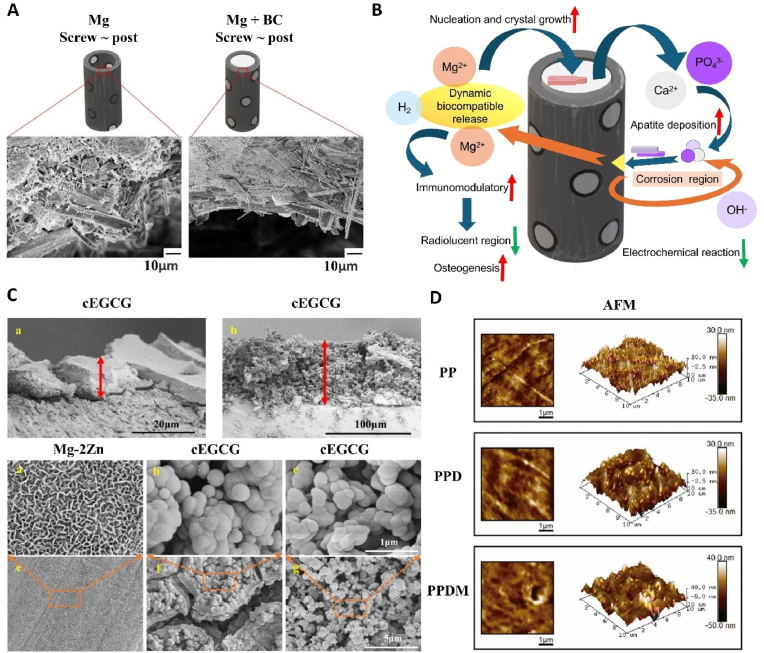
(A) Cross-sectional SEM scanning of screw component after degradation [181]. (B) Illustration of the underlying mechanism of tree-inspired Mg hybrid column orchestrating dynamic release of Mg ions to physiologically effective level and enhancing biomineralization in vivo [181]. (C) Mg-phenolic networks coating morphology characterization [189]. (D) Surface micromorphologies of the scaffolds [190].

As a representative bioactive element, magnesium is an important choice for surface modification of inert material [179]. The hybrid device, consisting of magnesium as a functional component and materials such as titanium alloy, tantalum alloy, and polyetheretherketone(PEEK) for mechanical support, achieves a balance between mechanical strength and biological activity [180]. Zu et al. [181] designed a special magnesium coating strategy that utilizes hollow magnesium metal as the outer layer, filled with bone cement inside, forming a bone implant with excellent corrosion resistance, mechanical strength, and osteogenic activity (Fig. 5A and B). However, how to control the release of Mg^2+^ is a challenging problem. The rapid degradation of the pure magnesium coating will lead to an explosive release of Mg^2+^. The results show that the current density of pure magnesium coating is high, and the pH value rises rapidly to about 11 within one day of the immersion test, reaching the maximum value [182]. It is necessary to improve the magnesium coating strategy. Mg-HA, formed by magnesium replacing calcium in HA, is a suitable form of magnesium applied to coatings. Mg and HA complement are complementary: the addition of Mg optimizes the mechanical properties and biological activity of HA, while HA help in the storage and controlled release of Mg^2+^ [183]. The preferential degradation of Mg determines the dynamic microporous structure of Mg-HA, which is beneficial to cell adhesion and bone ingrowth, and the released Mg^2+^ plays an osteoinductive role [184]. The coating methods for Mg-HA include electrochemical deposition, hydrothermal deposition, pulsed laser deposition and plasma spraying. Some studies have synthesized Mg-HA coating on the surface of titanium alloy scaffolds by electrochemical deposition, which verifies the rationality of the Mg-HA modification strategy and shows excellent osteogenic activity [185].

Magnesium salt as a coating for bone implants is another relatively simple and effective strategy, such as magnesium phosphate, magnesium sulfate, and magnesium silicate, which has a more stable release rate and avoid the adverse effects of hydrogen compared with magnesium. Besides the biological activity of Mg^2+^, magnesium salts also have obvious osteogenic effects, such as anionic phosphate and silicate [186,187]. There are many methods to prepare magnesium salt coatings, including in-situ growth, hydrothermal deposition and incorporation of organic polymers. Siahmard et al. [188] prepared forsterite (Mg_2_SiO_4_) coating on AZ31 magnesium alloy by sol-gel method. The relative stability of Mg_2_SiO_4_ as a composite magnesium salt significantly improves the corrosion resistance of the matrix. Metal phenol complexes can be formed due to the inherent binding force between phenols and metals. The magnesium phenol network coating designed in this way can be degraded stably and continuously, and shows quite good osteogenic activity in vivo experiments [189] (Fig. 5C). Adding adhesive to fix Mg^2+^ on the surface of the scaffold is also a desirable magnesium surface modification strategy, and the existence of adhesive is beneficial to the fixation and uniform distribution of Mg^2+^. Wei et al. [190]coated the porous PEEK scaffold with magnesium coating through the chelation of poly-dopamine (PDA) and Mg^2+^, and PDA helps the stable attachment and uniform distribution of Mg^2+^ on the surface of PEEK stents (Fig. 5D). Mg-PDA coating shows excellent osteogenesis angiogenesis and osteogenesis effects, especially in vivo experiments, which effectively enhance surface bone integration and bone in growth**.**

## 4 Multiple biological functions of magnesium

As one of the basic elements of the human body, magnesium participates in various physiological and metabolic reactions of the human body. Unlike inert material implants, magnesium-related implants positively affect the local microenvironment of bone defects. Magnesium exhibits high chemical reactivity in physiological environments, and its degradation primarily occurs through electrochemical reactions [191]. As magnesium degrades, a complex microenvironment containing Mg^2+^, OH^−^, and H_2_ is locally formed in the bone defect. Mg^2+^, one of the primary degradation products, plays a fundamental role in cellular physiological activities and participates in osteoblast-related cellular pathways. The alkaline environment created by OH^−^ is conducive to bone regeneration and bacteriostasis. Controlled H_2_ release is also a positive factor promoting osteogenesis. The positive osteogenic effects of Mg^2+^, OH^−^, and H_2_ are all concentration-dependent. The microenvironment formed by magnesium degradation exerts multiple effects, including osteogenic activity, angiogenic activity, immunomodulation, neuronal function, antibacterial properties, and antitumor effects [192]. Hence, it can modulate the osteogenic microenvironment under various complex pathological conditions, promoting bone regeneration through multiple dimensions. The various functions of magnesium in the process of bone repair and its molecular mechanism are discussed and analyzed further in this review.

### 4.1 Direct osteogenesis of magnesium

#### 4.1.1 Effect and mechanism on osteoblast-related cells

The microenvironment formed by magnesium degradation is conducive to various physiological activities of osteoblasts. Mg^2+^ as bioactive ion can maintain the activity of osteoblasts, enhance the migration, adhesion, and diffusion of bone marrow mesenchymal cells (BMSC) and, more importantly, activate the differentiation of BMSC [193]. Previous studies have shown that Mg^2+^ can increase the expression of osteogenic genes, such as alkaline phosphatase (ALP), osteopontin (OPN) and runt-related transcription factor 2 (Runx2) in osteoblasts [194]. The weakly alkaline environment formed by magnesium degradation can promote the proliferation and differentiation of osteoblasts and accelerate mineralization, with the most significant expression of osteoblastic proteins observed at a pH of 8.5 [195,196]. However, the direct mechanism of action of hydrogen gas on osteoblasts remains unclear.

**Fig. 6:**
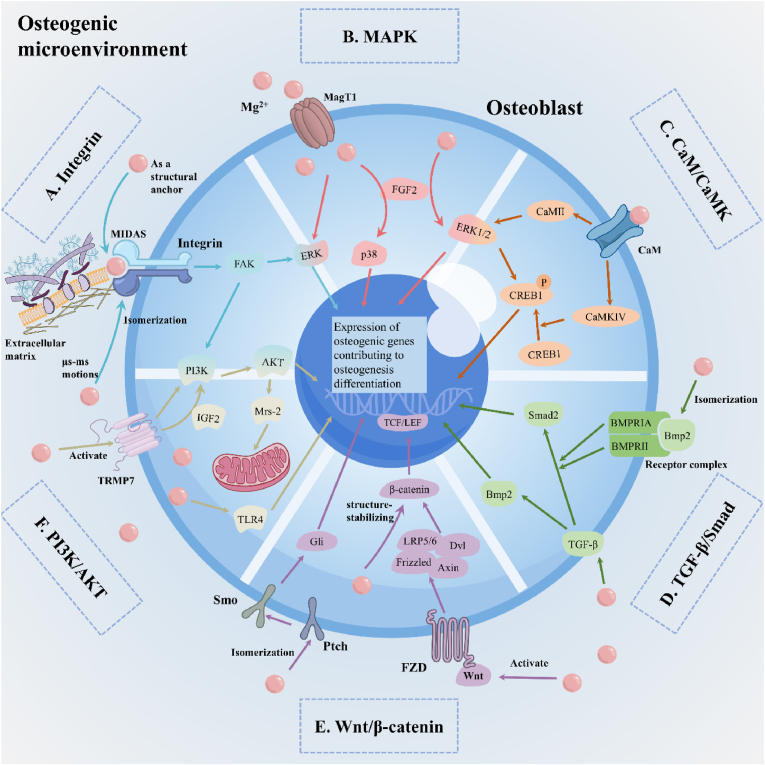
The signal Pathways Involved in Osteogenic effect of Mg^2+^.

Regarding the specific molecular mechanism, Mg^2+^ plays an important role in the function of integrin. Integrin, as a family of α/β heterodimer adhesion metalloprotein receptors, mediates the adhesion between ECM, and strengthens the adhesion between cytoskeleton and collagen through magnesium-dependent interaction. Mg^2+^ enhances the cell adhesion of BMSCs and promotes the subsequent osteogenic differentiation by up-regulating the expression of integrins α1, α2, α3, α5, and β1 [197,198]. Mg^2+^ plays a dual role in the integrin-collagen interaction. First, as a structural anchor, the metal ion-dependent adhesion site (MIDAS) on integrins, which regulates the functional characteristics of integrins, increases the affinity of integrins for ligands [199] (Fig. 6A). Collagen binds to the α1β1 integrin I domain by coordinating with Mg^2+^ on the MIDAS, thus anchoring the cell to the ECM and starting the downstream cascade reaction [200]. Second, according to the microsecond kinetic theory, Mg^2+^ activates the μs-ms exchange process to regulate the integrin-collagen interaction other than MIDAS, and produces a secondary Mg-binding substance that easily binds to collagen [199] (Fig. 6A). Furthermore, the release of Mg^2+^ activates integrin-focal adhesion kinase (FAK)/extracellular signal-regulated kinase (ERK) signal transduction and enhances osteogenic differentiation [201]. Besides Mg^2+^, the alkaline microenvironment has also been proven to promote osteogenesis by up-regulating the expression of integrins α2 and β1 [195].

The mitogen-activated protein kinase (MAPK) pathway is the downstream pathway of FAK, which is involved in bone development, remodeling, and metabolism. Studies have also shown that Mg^2+^ participates in two branch pathways, ERK1/2, and p38. Hong et al. found that Mg^2+^ also indirectly regulates the MAPK/ERK and MAPK/p38 pathways by increasing fibroblast growth factor (FGF) level [202] (Fig. 6B). Activation of ERK1/2 and p38 pathways enhanced the expression levels of downstream osteogenic genes such as c-fos, ALP and Runx2 [[203], [204], [205]].

The osteogenic effect of magnesium depends largely on its regulation of calcium homeostasis in the human body; therefore, the signal pathway related to calmodulin (CaM) is an important pathway for magnesium to promote bone formation. Mg^2+^ can bind to the Ca^2+^ binding site of CaM, while the binding of Mg^2+^ to CaM shows different biological activities from those of calcium ions. CaM can target and activate a variety of target proteins, among which Ca^2+^/calmodulin-dependent protein kinase (CaMK) family proteins are closely related to the osteogenic effect. As a physiological calcium antagonist, Mg^2+^ occupies the binding site of calcium ions in CaM by competitive inhibition, and activates the signal pathway of CaM-CaMKIV-cAMP response element binding protein 1 (CREB1) to up-regulate the expression of OPN [206] (Fig. 6C). Furthermore, Mg^2+^ is an essential cofactor of CaMKIV and CaMKIIα proteins and participates in its autophosphorylation process, thus activating CaMKII-ERK1/2-CREB/c-fos signal axis facilitates osteoblasts differentiation [207] (Fig. 6C).

A magnesium-rich microenvironment is beneficial to endogenous transforming growth factor-β1 and bone morphogenetic protein-2 (BMP-2), which contribute to the production of bone cytokines [208]. Mg^2+^ also acts directly on BMP-2 receptor, which triggers the Smad transcription signal pathway by enhancing BMP-2 receptor recognition, thus stimulating osteogenic differentiation and bone regeneration [208]. The modification of Mg^2+^ mediates the complex of BMP type IA receptor (BMPR IA) and BMP type II receptor (BMPR-II) on the cell surface and then significantly promotes the phosphorylation of Smad1/5/8. The modification of Mg^2+^ mediates the complex of BMPR IA and BMPR II on the cell surface and then significantly promotes the phosphorylation of Smad1/5/8 [209] (Fig. 6D).

The Wnt/β-catenin pathway is another contributing signaling pathway [210]. Mg^2+^ has been shown to have two important functions, namely, activating the Wnt pathway and stabilizing catenin (Fig. 6E). After being activated by Mg^2+^, Wnt protein combines with LRP5/6, Frizzled, Axin, and Dvl to form a complex, and β-catenin in the cytoplasm is phosphorylated and translocated into the nucleus, which combines with the T cell factor/lymphoenhancer family of transcription factors, thus promoting the transcription of downstream target genes to enhance the differentiation of osteoblasts [211,212]. In addition to the classical Wnt/β-catenin pathway, magnesium also acts on the Hedgehog pathway, which is an upstream signal pathway that replaces the Wnt pathway. Studies show that there are highly conserved binding sites of calcium, magnesium, and zinc on the surface of Hedgehog protein, which induces the formation of a high-affinity protein-protein interface between patched (Ptch) and the Hedgehog protein [213]. Mg^2+^ changes the conformation of Ptch through this mechanism, and then activates the hedgehog signal pathway [214]. Hedgehog signal transduction is composed of ligands and cell surface molecules (Ptch) and smoothened (Smo). The structural and conformational changes of Mg^2+^ weaken the inhibitory effect of Ptch on Smo; this promotes the activation of transcription factor glioma-associated oncogenes and activates the alternative Wnt pathway (Fig. 6E). Furthermore, studies have indicated that the degradation of magnesium metal in the body is accompanied by the deposition of Ca-P. Ca-P crystals benefit the physiological activity of chondrocytes and promote endochondral ossification by upregulating the Wnt3a/β-catenin signaling pathway [215].

Mg^2+^ also acts on the phosphoinositol 3-kinase (PI3K)/protein kinase B (Akt) signaling pathway to improve the proliferation and osteogenic differentiation activity of BMSC [216]. PI3K/Akt is a pathway involved in osteoblasts, immune regulation, angiogenesis, and nerve regeneration, especially for osteoblasts [217]. Mg^2+^ activates the PI3K/Akt pathway by mediating the upstream pathway of PI3K/Akt, such as transient-receptor-potential 7 (TRPM7) [218], Toll-like receptor 4 (TLR4) [219], and insulin-like growth factor (IGF)-2 [94]. Mg^2+^ activates TRPM7, which in turn phosphorylates PI3K downstream and activates the PI3K/Akt pathway [218] (Fig. 6F). Mg^2+^ also upregulates TLR4 in the body's microenvironment, which can directly induce osteogenic differentiation of BMSC and activate the PI3K/Akt pathway downstream [219] (Fig. 6F). Additionally, Mg^2+^ also acts on IGF. Magnesium indirectly stimulates osteogenesis by up-regulating IGF-2 to activate the PI3K/Akt signaling pathway [94] (Fig. 6F), and directly stimulates the expression of IGF-1 in osteoblasts to accelerate bone formation [220]. Phosphorylated Akt can promote the proliferation of osteoblasts and the expression of osteogenic related genes.

The effect of Mg^2+^ on osteoblasts is concentration-dependent, and an appropriate Mg^2+^ concentration can promote the proliferation and differentiation of BMSC [221]. However, magnesium deficiency will increase the risk of osteoporosis. High Mg^2+^ levels can change the crystal morphology of calcium nodules and inhibit collagen calcification by depleting calcium phosphate particles in mitochondria and accelerating autophagy, which has a negative effect on osteogenesis [222,223]. In terms of biocompatibility, Mg^2+^ becomes toxic to the BMSC of rats when its concentration exceeds 20 mM. In terms of osteogenic effect, some studies have shown that low extracellular concentrations of Mg^2+^ (0.1 mM) will inhibit the proliferation and differentiation of BMSC, and the concentration of Mg^2+^ of 2.5–5 mM is optimum to induce osteogenic differentiation of BMSC [224]. Another study showed that a Mg^2+^ concentration of 6–10 mM promoted the adhesion and proliferation of rat calvarial osteoblasts, and 10 mM Mg^2+^ significantly promoted the differentiation of osteoblasts. However, Mg^2+^ concentrations greater than 18 mM significantly inhibit the proliferation and differentiation of rat calvarial osteoblasts [225,226]. Overall, the safe concentration of Mg^2+^ is likely less than 20 mM, while the actual effective concentration of magnesium-related implants in the human body is between 2.5 mM and 10 mM. However, further experiments deserve to be conducted to study the optimal concentration range of Mg^2+^ for osteogenic differentiation of stem cells.

#### 4.1.2 Effect on osteoclasts

Magnesium is an important regulator of osteoclast [227]. Magnesium deficiency can affect the levels of vitamin D and parathyroid hormone in the human body, and promote the secretion of inflammatory cytokines to enhance the activity of osteoclasts [228]. The Mg^2+^, OH^−^, and H_2_ formed during magnesium degradation are all involved in regulating the physiological activities of osteoclasts.

Studies have shown that high concentrations of Mg^2+^ in the microenvironment regulates the activity of osteoclasts through vitamin D3, which affects the bone remodeling activity of Vitamin D3 [229]. However, appropriate concentration of Mg^2+^ in the microenvironment can indirectly inhibit the formation of osteoclasts through its anti-inflammatory effect. Studies have confirmed the feasibility of osteoclast depletion in the treatment of osteoporosis [227]. Although the osteoclast depletion mechanism of Mg^2^ in bone repair has not been extensively studied, its reported success in the treatment of osteoporosis provides ideas that can be explored further. The receptor activator of nuclear factor-kappa B ligand (RANKL)/RANK signal transduction pathway is the main pathway involved in osteoclast activity, and plays an irreplaceable role in the balance between osteoblasts and osteoclasts [230]. An appropriate concentration of Mg^2+^ acts on osteoblasts and stromal cells to produce a soluble bait receptor called osteoprotegerin (OPG), which inhibits the binding of RANKL to RANK on osteoclasts, thus preventing the differentiation and activation of osteoclasts [231]. Magnesium deficiency reduces OPG secretion and alleviates the inhibition of the RANK/RANKL pathway [228].

The activity of osteoclasts is pH-dependent, reaching its peak bone resorption activity under acidic conditions at pH 6.8 [232]. However, the generation of OH^−^ creates a locally weak alkaline environment at the site of bone defects, which attenuates the activity of osteoclasts [233]. Notably, H_2_ significantly inhibits the proliferation of osteoclasts, suppresses the expression of osteoclast-related genes and proteins, and promotes their apoptosis [234].

### 4.2 Immunoregulation

Immune regulation plays an irreplaceable role in the healing process of bone defects [235]. Immune cells, especially macrophages, play an irreplaceable role in the process of osteogenesis. They produce a variety of cytokines and growth factors to promote cell aggregation and bone differentiation at the site of bone defects.

The degradation of magnesium triggers the secretion of the proinflammatory factor interleukin (IL)-6 in the body, which promotes the recruitment and adhesion of neutrophils, initiating an immune response [236]. Magnesium can coordinate the pro-inflammatory and anti-inflammatory reactions at the site of bone defects by participating in the sequential activation of M1 and M2 phenotypic macrophages, which is beneficial to the regulation of osteogenic differentiation in the bone defect region. It has been found that magnesium implants tend to polarize macrophages to the M1 type on the first day of implantation [237]. This is due to an increase in pH in the microenvironment of bone defects managed using Mg^2+^ caused by the degradation zone around the implant and the release of Mg^2+^, which regulates the aggregation of proteins, such as fibrinogen, on the implant surface. Furthermore, the activation of M1 macrophages through the up-regulation of αM and TLR facilitates the recognition of foreign bodies and damaged cells and recruits Myeloid differentiation factor 88, Toll/IL-1R domain-containing adaptor molecule (Ticam)-1, and Ticam-2 [238]. However, unlike typical polarized M1 macrophages, the expression of the M2 marker gene C-C motif chemokine ligand 24 was up-regulated under Mg^2+^ induction [237]. Early M1 macrophages are important for the initiation of osteogenic repair; however, the persistence of an inflammatory reaction will aggravate the tissue damage of bone defects. The advantages of magnesium extend beyond bone regeneration by activating inflammatory reactions; it also initiates bone and surrounding repair, even after the inflammatory reaction has terminated. Therefore, Mg^2+^ induces autophagy in macrophages by inhibiting the Nuclear Factor-κB (NF-κB) pathway, which is closely related to the inflammatory response, promoting the polarization of macrophages from M1 to M2, down-regulating the pro-inflammatory response, and up-regulating the anti-inflammatory response [239,240]. Additionally, Mg^2+^ also inhibit the inflammatory response of macrophages by mediating TRPM7 ion channels [241]. Not only does the macrophage indirectly regulate osteogenesis by modulating the immune microenvironment, but it also directly affects osteoblast-related cells. Studies have shown that macrophages are closely related to the physiological activities of periosteum-derived stem cells (PDSCs). Mg degradation products enhance the osteogenesis of PDSCs by promoting M2 macrophage polarization and IL-10 secretion, thereby activating the JAK1-STAT3 pathway [242]. Studies have indicated that high concentrations of Mg^2+^ have a significant effect on macrophages. Xie et al. demonstrated that a Mg^2+^ concentration of 43.3 mM significantly upregulates M1-related genes, promoting M1 macrophage polarization [243]. Sun et al. demonstrated that a Mg^2+^ concentration of less than 70 mM is optimal for M2 macrophage polarization [244].

In addition to its role in innate immune responses, magnesium also acts on the ensuing adaptive immune responses. Magnesium can promote the maturation of antigen-presenting cells and initiate the adaptive immune system. Magnesium is an important cofactor in immunoglobulin (Ig) synthesis, immune cell adhesion, IgM lymphocyte binding, macrophage response to lymphatic factor, and immune activation processes, all of which are closely related to the functions of T and B cells [192].

Apart from its role in osteoblasts, magnesium also mediates angiogenesis and neural regeneration through immune modulation. Some studies have shown that Mg^2+^ protects vascular endothelial cells by blocking P2X7 receptor and inhibiting lipopolysaccharide-induced inflammatory reaction, which shows that the immunomodulatory effect of Mg^2+^ is beneficial to the protection and regeneration of blood vessels [245]. Furthermore, as a foundation for neurotransmission and the maintenance of ionic homeostasis, Mg regulates the homeostasis of the nervous system through immune modulation. Magnesium can alleviate inflammatory responses in the nervous system by antagonizing calcium and inhibiting substance P and [246]. Furthermore, divalent cations, represented by Mg^2+^, have also been demonstrated to stimulate bone sensation by promoting the secretion of prostaglandin E2 from macrophages, thereby downregulating sympathetic tone to form new bone [247].

### 4.3 Angiogenesis of magnesium

**Fig. 7:**
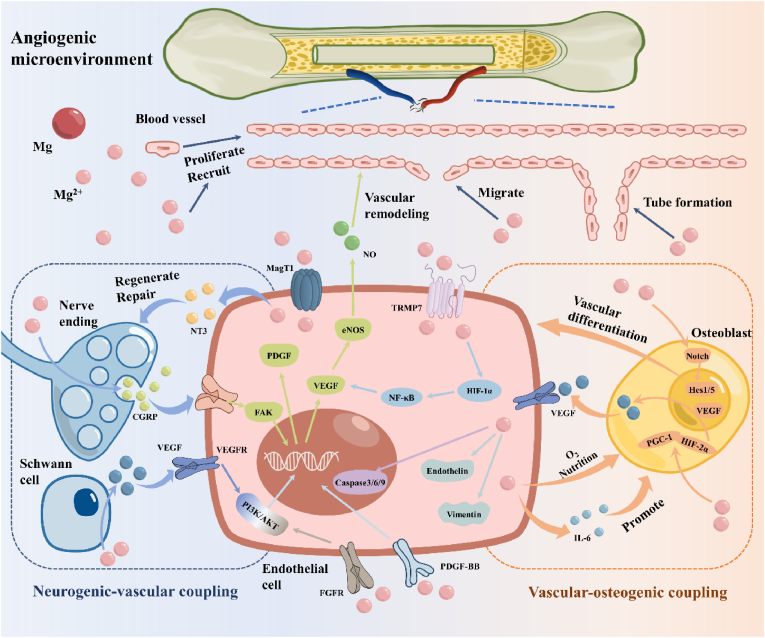
Mechanism of action of Mg^2+^ in vascular remodeling and bone defect repair.

The angiogenic effect of magnesium is another highlight of its application in the treatment of bone defects [248]. The ingrowth of blood vessels is a prerequisite for bone regeneration. Microvessels provide oxygen and nutrition for bone tissue growth and repair, and transport necessary osteogenesis-related growth factors to regulate osteogenesis [249]. The activity of angiogenesis in bone defects and the integrity of vascular network reconstruction determine the speed and effect of bone regeneration [250]. Owing to the excellent angiogenesis effect of magnesium, numerous researchers have successfully added bioactive Mg^2+^ to bone implants to achieve vascularized bone regeneration. This chapter summarizes the angiogenesis mechanism of Mg^2+^, as well as neural-vascular coupling and vascular-osteogenic coupling (Fig. 7).

As mentioned above, the release of Mg^2+^ up-regulates TRPM7, promotes the influx of Mg^2+^ in bone marrow macrophages, and creates a local immune microenvironment that is conducive for angiogenesis [136]. In addition to the indirect effect of changing the local microenvironment in the bone defect area, Mg^2+^ ions can also recruit vascular endothelial cells and are recognized by endothelial cells, which can stimulate the production of reactive oxygen species(ROS) [251]. Magnesium influx and ROS synergistically induce the up-regulation of vascular endothelial growth factor (VEGF) and FGF expression in endothelial cells, which increases the sensitivity of endothelial cells to proliferation and migration signals [252]. The release of Mg^2+^ upregulates the transcription level of angiogenesis-related cytokines and endothelial cell markers, and downregulates the expression level of endothelial cell apoptosis-related genes [136]. Mg^2+^ promotes hypoxia-inducible factor (HIF)-1α expression by stimulating the generation of ROS and endothelial nitric oxide synthase (eNOS)-related pathways [253]. HIF-1α is a transcription factor known to regulate VEGF expression through NF-κB activation, and its up-regulation stimulates VEGF transcription [254]. VEGF plays a key role in endothelialization, and can stimulate the production of nitric oxide (NO) in vascular media through the PI3K/Akt/eNOS pathway [253]. Mg^2+^ also induces the formation of platelet-derived growth factor-BB in the microenvironment and contributes to the formation of CD31hiEmcnhi blood vessels in bone remodeling [255]. According to Liu et al.'s study, the Mg^2+^ concentration suitable for various physiological activities of endothelial cells ranges from 1 to 5 mM, particularly at 5 mM [256].

As shown in Fig. 7, Mg^2+^ participates in vascular-osteogenic coupling in bone regeneration. Mg^2+^ causes high expression of magnesium transporter-1 in BMSC, which accelerates the influx of Mg^2+^ in BMSC; furthermore, Mg^2+^ induces angiogenesis and differentiation of BMSC through Notch signal transduction in cells [224,257]. The release of Mg^2+^ activates the Notch signaling pathway, up-regulates downstream genes hairy and enhancer of split (Hes)-1 and Hes-5, and angiogenesis-related genes Hif-1α and eNOS, thus promoting angiogenesis and differentiation of BMSCs. Mg^2+^ has been shown to stimulate the secretion of VEGF by activating HIF-2α and proliferator-activated receptor γ coactivator 1α in BMSC [258]. Studies have shown that a Mg^2+^ concentration of 5 mM is optimal for angiogenesis and the differentiation of BMSC [257]. Besides relying solely on blood vessels to provide nutrition and oxygen, Mg^2+^ can also stimulate endothelial cells to secrete osteogenic factors, such as IL-6, to promote bone regeneration [259].

Nervous and vascular activities of bones are inseparable. Fig. 7 shows the mechanism through which Mg^2+^ participates in neurogenic-vascular coupling. Studies have shown that Mg^2+^ in the microenvironment can recruit BMSCs to bone defects and drive BMSCs to differentiate into nerve cells. BMSC-derived nerve cells and Mg^2+^ can synergistically promote angiogenesis [260]. Mg^2+^ targets the dorsal root ganglion (DRG), induces calcitonin gene-related peptide (CGRP) release, and activates the CGRP-FAK-VEGF pathway, which is a representative pathway of bone nerve-blood vessel coupling [261]. Schwann cells (SCs) can secrete VEGF after being induced by Mg^2+^ [20]. The establishment of a bone vascular network subsequently leads to the secretion of neurotrophic factors to promote the regeneration of peripheral nerves. Endothelial cells can synthesize and release neurotrophic factor 3 and artemisinin; therefore, it is beneficial to the repair of nerves in bone defect areas and further neuroosteogenesis [20]. Overall, Mg^2+^ participates in the neurovascular coupling of bone system, which is helpful to rebuild the neurovascular network of the periosteum and bone marrow cavity, thus greatly accelerating the healing and repair of large bone defects.

### 4.4 Neurogenesis of magnesium

In the normal physiological state, bones have abundant nervous tissues, including peripheral sensory nerve fibers and sympathetic nerve fibers, which can stimulate and regulate osteogenesis [262]. In a study of biomaterials for bone implants, the establishment of simulated natural bone tissue and vascular networks has always been a source of concern; however, the reconstruction of neural networks around bones was ignored [20]. Periosteum, as an important structure of osteogenesis, has a rich neural network in its outer fibrous layer, which provides nutrition and signal molecules for bone tissue [263]. Bone metabolism and bone formation are monitored and regulated by sensory nerves, motor nerves, and various neurogenic factors (such as neuropeptide Y, SP, and CGRP) [[264], [265], [266]]. Furthermore, the central nervous system (CNS) also mediates bone formation through the involvement of serotonin, Sema3A, and brain-derived neurotrophic factor in bone homeostasis and regeneration [266].

**Fig. 8:**
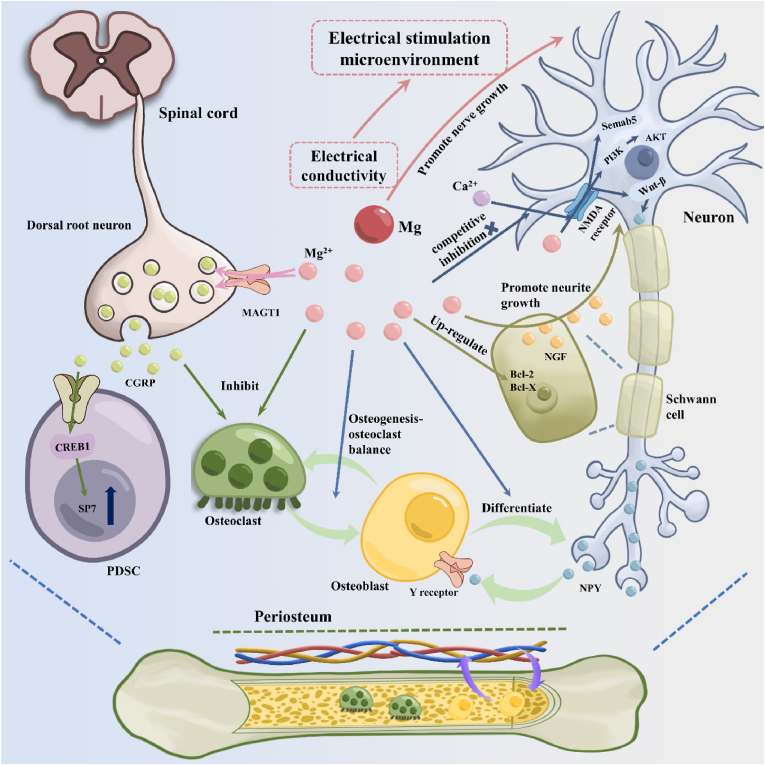
The mechanism of Mg and Mg^2+^ in neuroosteogenesis.

Studies have shown that Mg^2+^ regulates the process of bone repair by promoting the germination and growth of nerves [267]. Fig. 8 demonstrates the neuro-bone regulation mechanism of Mg and Mg^2+^ in the microenvironment of bone defects, and illustrates that Mg^2+^ indirectly regulates bone regeneration by acting on nerve cells, such as DRG and SCs. Magnesium itself is an excellent conductor, and its electrical conductivity provides an electrical stimulation environment for nerve regeneration and enhances neurite regeneration [268]. In the nervous system, Mg^2+^ plays a key role in maintaining calcium homeostasis. As the second messenger, Mg^2+^ inhibits the influx of Ca^2+^ by blocking the ion channel of the N-methyl-D-aspartate receptor, thus restraining the neurotoxicity of Ca^2+^ [269] (Fig. 8). The antagonism of calcium signal transduction makes Nod-like receptor protein 3 inflammatory corpuscles and IL-1β up-regulated, which has the effect of inhibiting nerve cell apoptosis [270]. In addition to the effect on calcium homeostasis, the lack of Mg^2+^ will also lead to the production of ROS and mitochondrial damage, reduce the activity of superoxide dismutase, and eventually lead to the death of neurons [271]. Mg^2+^ can also activate the PI3K/Akt signal pathway and semaphorin 5b to promote neurite growth [269] (Fig. 8).

The Wnt signaling pathway is a crucial mechanism in neural regeneration. Mg^2+^ regulate SCs proliferation, migration, and phenotypic transformation towards repair through the Wnt/β-catenin pathway [272]. Mg^2+^ also can reduce the apoptosis of SCs by promoting the expression of B-cell lymphoma (bcl)-2 and bcl-X [273]. Similarly, Mg^2+^ induces SCs to secrete nerve growth factor, which promotes the growth of nerve axons [263]. Mg^2+^ also targets DRG cells, which induces the release of CGRP [274]. The release of CGRP induced by Mg^2+^ stimulates the increase of osterix levels in periosteal stem cells, thus promoting bone regeneration [275] (Fig. 8). CGRP also acts as a direct inhibitor of osteoclasts and reduces bone resorption [20]. Generally, the neuroprotective effect of Mg^2+^ and the secretion of neuropeptide positively regulate the osteogenic recovery of bone defects and the repair of peripheral nerve injuries. Zhang et al. [274] demonstrated that Mg^2+^ promote CGRP-mediated osteogenic differentiation by stimulating the peripheral cortical nerves of the femur and ipsilateral DRG, through the implantation of ultrapure magnesium intramedullary pins into the distal femur of rats.

The brain-bone axis represents a crucial aspect of neuroskeletal regulation, encompassing bidirectional communication between the CNS and bone metabolism [276]. The CNS engages in bone metabolism and remodeling through multiple neural pathways and neurotransmitters. Studies have indicated that Mg^2+^ plays an irreplaceable role in the brain-bone axis. Mg^2+^ contributes to the homeostasis of the CNS, coordinating the interaction between the CNS and bone metabolism to maintain normal physiological activities of bone metabolism. However, the specific biological effects of Mg^2+^ on the CNS's regulation of bone remain elusive. We hypothesize that the Wnt pathway is one of the mechanisms by which Mg^2+^ regulates the CNS to promote bone formation, as the expressions of Wnt-1 and Wnt-7 are upregulated after denervation during ectopic bone regeneration [277].

### 4.5 Anti-tumor effect of magnesium

The treatment of bone tumors has changed from amputation to limb salvage surgery. However, the reconstruction of bone defects after tumor resection is still a challenging problem, and postoperative bone reconstruction puts forward requirements for implants, including good mechanical properties, osteogenic properties, and inhibition of bone tumors. Traditional implants can provide mechanical support for bone defects and promote bone activity, but they cannot remove recurrent tumor cells. Magnesium has been used in bone defect reconstruction after tumor resection and has achieved good results. Some studies have shown that magnesium can prevent the progression of bone tumors by promoting tumor cell apoptosis, inhibiting tumor cell proliferation and invasion, and improving the tumor immune environment [278]. Magnesium exerts its anti-tumor effect through its thermal effect and its degradation products, Mg^2+^, OH^−^, and H_2_.

**Fig. 9:**
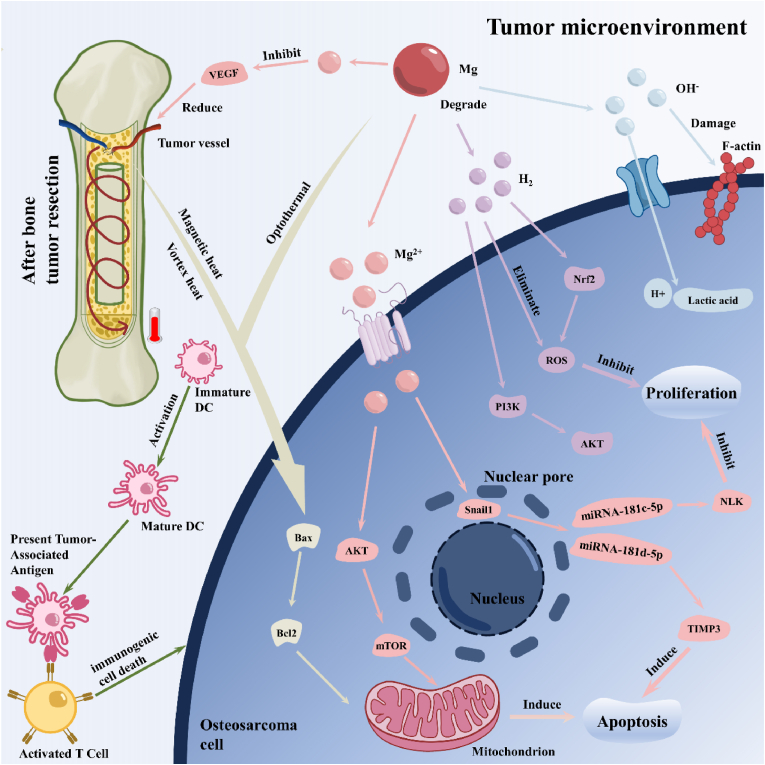
Application of magnesium and its degradation products in bone defect after bone tumor resection and its anti-bone tumor mechanism.

Mg^2+^ induces mitochondrial apoptosis in tumor cells through magnesium-dependent Akt/mammalian target of rapamycin/bax related pathways [279]. Furthermore, Mg^2+^ promotes the phosphorylation and nuclear transfer of zinc finger protein Snail 1, and then activates the parallel anti-tumor signal pathways downstream of miRNA-181d-5p-TIMP3 and miRNA-181c-5p-NLK to inhibit the proliferation and induce apoptosis of osteosarcoma cells (Fig. 9). The concentration range of Mg^2+^ that exerts anti-tumor effects is 10–20 mM. Within this concentration range, normal bone cells can survive and perform physiological activities normally [280].

The pH of the tumor microenvironment is lower than that of normal tissues, and tumor cells are most physiologically active at a pH of 6.8, rather than the 7.3 found in normal cells [281]. The OH^−^ released during magnesium degradation disrupt the acidic tumor microenvironment and maintain the Warburg effect, which is essential for tumor metabolism [282,283]. Li et al. stained the skeleton of osteosarcoma Mg-63 cells seeded on the surface of Mg material and found that an alkaline environment could damage the skeleton F-actin of osteosarcoma cells, thus inhibiting the adhesion of osteosarcoma cells on the surface of Mg material [282] (Fig. 9).

The H_2_ released by magnesium degradation has antioxidant and anti-tumor properties. H_2_ upregulates the expression of tumor suppressor protein P53, thus activating the mitochondrial related apoptosis pathway [58]. H_2_ also acts on PI3K/Akt signaling pathway to inhibit the proliferation of tumor cells. The tumor microenvironment is often accompanied by oxidative stress, which is due to excessive free radicals produced by malignant tumors, resulting in oxidative stress in local areas [284]. The reduction characteristics of H_2_ and up-regulation of nuclear factor erythroid-2 related factor 2 expression can effectively eliminate ROS and other free radicals, thus inhibiting the growth and migration of tumor cells [285,286] (Fig. 9).

The anti-tumor performance of elemental magnesium depends on its excellent thermal conductivity and photothermal effect, which can kill bone tumor cells through thermal conduction [287,288]. Research has shown that tumor cells undergo rapid necrosis and clearance at temperatures between 45 and 48 °C [289]. Under 808-nm near-infrared light irradiation, the temperature of magnesium rises to 45–55 °C, enabling selective cell ablation of osteosarcoma Saos-2 cells [287]. Ge et al. [290] used the eddy current thermal effect and magnetocaloric effect of magnesium to design magnesium rods. Magnetic hyperthermia mediated by it directly kills osteosarcoma and causes the immunogenic cell death of osteosarcoma cells caused by the eddy heat effect (Fig. 9).

Materials and drugs targeting tumors often damage normal cells while killing tumor cells. Magnesium is a potential material that possesses both antitumor and osteogenic effects. Through its slow degradation characteristics, magnesium-related implants exhibit early antitumor effects after implantation and later osteogenic effects [291].

### 4.6 Antimicrobial effect

Magnesium has diverse antibacterial mechanisms and a wide range of bactericidal spectrums. As mentioned above, the degradation of magnesium induced the infiltration of macrophages, which gathered at the implantation site [292]. Mg^2+^ produced by degradation activates M1-type macrophages with pro-inflammatory and antibacterial properties. Pro-inflammatory factors, such as TNFα, IL1β, IL-6, IL-8, and MCP1, secreted by M1 macrophages have preventive and therapeutic effects on infection. Other studies have shown that the presence of bacteria will prompt macrophages to switch to the classic activated M1 type [293]. Moreover, Mg^2+^ can induce the production of ROS, thus destroying the cell wall and cell membrane of microorganisms and causing their death [294]. Some studies have also shown that RAW cells cultured in a high concentration Mg^2+^ environment can secrete inducible nitric oxide synthase (iNOS). NO derived from iNOS is an important weapon to kill microorganisms, which can induce neutrophil chemotaxis and migration to infected bone defects [295]. This provides an idea for its application in infectious bone defects.

**Fig. 10:**
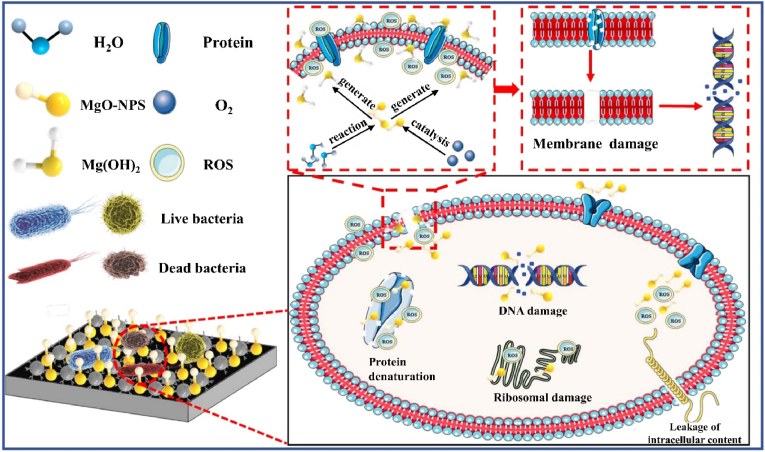
Antimicrobial mechanism of compact HA/MgO nano-composite coating [294].

The bacteriostatic mechanism of Mg and MgO nanoparticles can be developed from the direct bacteriostatic effect and the indirect bacteriostatic effect of degradation to produce Mg^2+^. Mg-NPs and MgO-NPs can directly kill microorganisms. The antibacterial mechanism of MgO-NPs is that the electrons generated during the hydration of MgO-NPs and ROS induce lipid peroxidation of the bacterial cell membrane (Fig. 10). In particular, MgO has particle aggregation, which can form a local high-concentration MgO state to obviously enhance its killing effect on microorganisms [294]. Therefore, it is necessary to construct dense and evenly distributed MgO-NPs and control their carrying capacity and release rate [296]. The local degradation of Mg-NPs and MgO-NPs forms an alkaline microenvironment, which is not conducive to the survival and reproduction of microorganisms [293]. Mg^2+^ generated by degradation induces iNOS to produce high levels of NO to enhance local immunity and inhibit microorganisms through lipid peroxidation [295,297]. More importantly, studies have shown that both MgO-NPs and Mg^2+^ can induce ROS to destroy bacterial cell walls and cell membranes [294,297].

## 5 Clinical study of magnesium-related implants

Magnesium-related implants hold significant potential in the treatment of bone defects. However, most studies are still in the preclinical testing phase using animal experiments. Based on promising results from various animal studies, some clinical investigations have been initiated. Currently, the clinical application of magnesium-related implants is primarily focused on areas with lower load-bearing requirements, such as screws for fracture fixation. In 2013, the MgYREZr alloy screw, reported as the world's first biodegradable screw, was utilized for fixation in hallux valgus surgical correction. Studies have indicated that within the healing time frame of hallux valgus, the prognosis of the magnesium-based screw group showed no significant difference compared to the traditional titanium screw group, and no rejection, inflammation, or osteolysis was observed [298]. As a highly safe biomaterial, high-purity magnesium has also been prioritized for clinical application. Studies applying high-purity magnesium screws in bone graft fixation for femoral head necrosis have demonstrated excellent osseointegration and degradation properties [299]. Recent years have seen rapid advancements in basic research and clinical translational studies of JDBM magnesium alloy. Xie et al. [95] investigated the fixation effect of JDBM magnesium alloy coated with Ca-P in ankle fractures, proving that JDBM fixation facilitates good fracture reduction and functional recovery. The successful application of the aforementioned magnesium-based screws illustrates the promising clinical translation prospects of magnesium-related implants. However, the current clinical application scope of magnesium-related implants remains limited, and clinical research on high-load-bearing bone implants required for large bone defects and joint replacement surgeries is still in its infancy. Furthermore, there is a lack of uniform evaluation criteria for magnesium-based bone implants. Their biodegradability necessitates the establishment of new standards distinct from traditional implants. Additionally, due to their relatively short clinical application time, there is a scarcity of long-term clinical data to adequately assess their safety and efficacy. The clinical translation of magnesium-related bone implants is based on material optimization and continuous innovation driven by clinical needs. This approach aims to design implant materials that are safe, mechanically strong, and have appropriate degradation rates. Additionally, a standardized preclinical evaluation system needs to be established to comprehensively assess the performance and potential risks of magnesium-related implants from multiple perspectives. Large-scale, multicenter clinical trials should be conducted based on reasonable preclinical evaluations to accelerate the clinical translation of magnesium-related bone implants. The clinical translation research of magnesium-related implants is a long and arduous task, but its bright future is worth anticipating.

## 6 Conclusion

Reconstruction of bone defects has long been a challenging orthopedic clinical problem. Magnesium is a bioactive metal that participates in the process of bone metabolism and bone regeneration. Magnesium-related implants, characterized by their unique degradation properties and mechanical performance, are frequently employed in research for the treatment of bone defects. Based on the advantages of magnesium and its physical properties and biological activity, the application modes of magnesium are divided into magnesium-based scaffolds and magnesium functionally modified scaffolds. In terms of its physical properties, magnesium's biodegradability and elastic modulus are close to those of cortical bone. However, its application is limited by its rapid degradation rate and insufficient mechanical strength. These disadvantages can be ameliorated by using modification strategies such as alloying and surface modification. Currently, a variety of bone implants based on magnesium have been studied, and excellent feedback has been obtained. However, the biological activity of magnesium makes it an excellent functional modification material, and endows inert base materials with excellent osteogenic activity. Magnesium is involved in all stages and directions of bone repair, which can regulate the balance between osteogenesis and osteoclast, improve the immune microenvironment, and indirectly form bone through vascularization and neurogenesis. Additionally, magnesium has certain anti-tumor and antibacterial effects on bone defects and infectious bone defects after tumor resection. The application strategies and modification methods for magnesium are also versatile, including the application of magnesium nano-ions, the loading of microspheres, hydrogels, and coatings.

Although magnesium has been widely used as a base material and a functional modification material, its application is faced with some drawbacks that need to be solved. There are a wide range of alloying options for magnesium, including combinations to control alloying elements or doping ratios; however, there is still no authoritative magnesium alloy system for orthopedic applications. Furthermore, the specific mechanism of Mg as a metal and its degradation products in the process of bone regeneration is still not fully understood, and most of the existing data comes from in vitro experimental results; therefore, it is impossible to construct a complete theoretical system. There is still a need to explore the mechanism of Mg's action in the complex microenvironment of bone defects in vivo, while also considering the bioactivity of other alloy elements, to construct an optimal alloy system and provide an opportunity for its clinical translation.

## CRediT authorship contribution statement

**Mijia Tao:** Writing – original draft, Investigation, Data curation, Conceptualization. **Yutao Cui:** Writing – original draft, Investigation. **Shicai Sun:** Validation, Methodology. **Yan Zhang:** Validation, Data curation. **Jianli Ge:** Validation, Data curation. **Wen Yin:** Visualization, Software. **Peng Li:** Visualization, Software. **Yanbing Wang:** Writing – review & editing, Supervision, Funding acquisition, Conceptualization.

## Declaration of competing interest

The authors declare that they have no known competing financial interests or personal relationships that could have appeared to influence the work reported in this paper.

## Data Availability

No data was used for the research described in the article.

## References

[1] Ho-Shui-Ling A., Bolander J., Rustom L.E., Johnson A.W., Luyten F.P., Picart C. (2018). Bone regeneration strategies: engineered scaffolds, bioactive molecules and stem cells current stage and future perspectives. Biomaterials.

[2] Lin H., Wang X., Huang M., Li Z., Shen Z., Feng J., Chen H., Wu J., Gao J., Wen Z. (2020). Research hotspots and trends of bone defects based on Web of Science: a bibliometric analysis. J. Orthop. Surg. Res..

[3] Zhi W., Wang X., Sun D., Chen T., Yuan B., Li X., Chen X., Wang J., Xie Z., Zhu X. (2022). Optimal regenerative repair of large segmental bone defect in a goat model with osteoinductive calcium phosphate bioceramic implants. Bioact. Mater..

[4] Sohn H.-S., Oh J.-K. (2019). Review of bone graft and bone substitutes with an emphasis on fracture surgeries. Biomater. Res..

[5] Rogers G.F., Greene A.K. (2012). Autogenous bone graft: basic science and clinical implications. J. Craniofac. Surg..

[6] Tournier P., Guicheux J., Pare A., Veziers J., Barbeito A., Bardonnet R., Corre P., Geoffroy V., Weiss P., Gaudin A. (2021). An extrudable partially demineralized allogeneic bone paste exhibits a similar bone healing capacity as the "gold standard" bone graft. Front. Bioeng. Biotechnol..

[7] Cao S., Zhao Y., Hu Y., Zou L., Chen J. (2020). New perspectives: in-situ tissue engineering for bone repair scaffold. Composites Part B-Engineering.

[8] Glasdam S-M, Glasdam S, Peters GH: The importance of magnesium in the human body: a systematic literature review**.** In Adv. Clin. Chem., Vol 73. *Volume* 73. Edited by Makowski GS2016: 169-193: *Advances in Clinical Chemistry*]. 10.1016/bs.acc.2015.10.002 26975973

[9] Chen Y., Sheng W., Lin J., Fang C., Deng J., Zhang P., Zhou M., Liu P., Weng J., Yu F. (2022). Magnesium oxide nanoparticle coordinated phosphate-functionalized chitosan injectable hydrogel for osteogenesis and angiogenesis in bone regeneration. ACS Appl. Mater. Interfaces.

[10] Mammoli F., Castiglioni S., Parenti S., Cappadone C., Farruggia G., Iotti S., Davalli P., Maier J.A.M., Grande A., Frassineti C. (2019). Magnesium is a key regulator of the balance between osteoclast and osteoblast differentiation in the presence of vitamin D-3. Int. J. Mol. Sci..

[11] Zhao D., Witte F., Lu F., Wang J., Li J., Qin L. (2017). Current status on clinical applications of magnesium-based orthopaedic implants: a review from clinical translational perspective. Biomaterials.

[12] Lin T., Wang X., Jin L., Li W., Zhang Y., Wang A., Peng J., Shao H. (2021). Manufacturing of porous magnesium scaffolds for bone tissue engineering by 3D gel-printing. Mater. Des..

[13] Rider P., Kacarevic Z.P., Elad A., Tadic D., Rothamel D., Sauer G., Bornert F., Windisch P., Hangyasi D.B., Molnar B. (2022). Biodegradable magnesium barrier membrane used for guided bone regeneration in dental surgery. Bioact. Mater..

[14] Xie K., Wang N., Guo Y., Zhao S., Tan J., Wang L., Li G., Wu J., Yang Y., Xu W. (2022). Additively manufactured biodegradable porous magnesium implants for elimination of implant-related infections: an in vitro and in vivo study. Bioact. Mater..

[15] Yao R., Zhao Y., Han S., Shan R., Liu L., Sun Y., Yao X., Wang X., Hang R. (2023). Microstructure, mechanical properties, in vitro degradation behavior and in vivo osteogenic activities of Zn-1Mg-beta-TCP composites for bone defect repair. Mater. Des..

[16] Wei X., Zhou W., Tang Z., Wu H., Liu Y., Dong H., Wang N., Huang H., Bao S., Shi L. (2023). Magnesium surface-activated 3D printed porous PEEK scaffolds for in vivo osseointegration by promoting angiogenesis and osteogenesis. Bioact. Mater..

[17] Lin S., Yin S., Shi J., Yang G., Wen X., Zhang W., Zhou M., Jiang X. (2022). Orchestration of energy metabolism and osteogenesis by Mg2+ facilitates low-dose BMP-2-driven regeneration. Bioact. Mater..

[18] Gao P., Fan B., Yu X., Liu W., Wu J., Shi L., Yang D., Tan L., Wan P., Hao Y. (2020). Biofunctional magnesium coated Ti6Al4V scaffold enhances osteogenesis and angiogenesis in vitro and in vivo for orthopedic application. Bioact. Mater..

[19] Ning T., Yang F., Chen D., Jia Z., Yuan R., Du Z., Liu S., Yu Y., Dai X., Niu X., Fan Y. (2022). Synergistically detachable microneedle dressing for programmed treatment of chronic wounds. Adv. Healthcare Mater..

[20] Xu Y., Xu C., He L., Zhou J., Chen T., Ouyang L., Guo X., Qu Y., Luo Z., Duan D. (2022). Stratified-structural hydrogel incorporated with magnesium-ion-modified black phosphorus nanosheets for promoting neuro-vascularized bone regeneration. Bioact. Mater..

[21] Huang C., Li D., Song J., Chen K., Wang X., Zhao F., Gu X., Xie X., Fan Y. (2023). Dual-functional coatings on magnesium alloys: enhancing corrosion behavior under stress and osteogenic effect in osteoporotic rats. Appl. Mater. Today.

[22] Chen Y., Sun Y., Wu X., Lou J., Zhang X., Peng Z. (2022). Rotator cuff repair with biodegradable high-purity magnesium suture anchor in sheep model. Journal of Orthopaedic Translation.

[23] Wu S., Jang Y.-S., Lee M.-H. (2021). Enhancement of bone regeneration on calcium-phosphate-coated magnesium mesh: using the rat calvarial model. Front. Bioeng. Biotechnol..

[24] Kopp A., Fischer H., Soares A.P., Schmidt-Bleek K., Leber C., Kreiker H., Duda G., Kroeger N., van Gaalen K., Hanken H. (2023). Long-term in vivo observations show biocompatibility and performance of ZX00 magnesium screws surface-modified by plasma-electrolytic oxidation in Gottingen miniature pigs. Acta Biomater..

[25] Li D., Zhang D., Yuan Q., Liu L., Li H., Xiong L., Guo X., Yan Y., Yu K., Dai Y. (2022). In vitro and in vivo assessment of the effect of biodegradable magnesium alloys on osteogenesis. Acta Biomater..

[26] Dou C., Zhang M., Ren D., Ji H., Yi Z., Wang S., Liu Z., Wang Q., Zheng Y., Zhang Z., Yang R. (2023). Bi-continuous Mg-Ti interpenetrating-phase composite as a partially degradable and bioactive implant material. J. Mater. Sci. Technol..

[27] Bonithon R., Kao A.P., Fernandez M.P., Dunlop J.N., Blunn G.W., Witte F., Tozzi G. (2021). Multi-scale mechanical and morphological characterisation of sintered porous magnesium-based scaffolds for bone regeneration in critical-sized defects. Acta Biomater..

[28] Peron M., Bertolini R., Cogo S. (2022). On the corrosion, stress corrosion and cytocompatibility performances of ALD TiO_2_ and ZrO_2_ coated magnesium alloys. J. Mech. Behav. Biomed. Mater..

[29] Chen L., Sheng Y., Zhou H., Li Z., Wang X., Li W. (2019). Influence of a MAO plus PLGA coating on biocorrosion and stress corrosion cracking behavior of a magnesium alloy in a physiological environment. Corros. Sci..

[30] Munir K., Lin J., Wen C., Wright P.F.A., Li Y. (2020). Mechanical, corrosion, and biocompatibility properties of Mg-Zr-Sr-Sc alloys for biodegradable implant applications. Acta Biomater..

[31] Martin V., Garcia M., Montemor MdF., Fernandes J.C.S., Gomes P.S., Fernandes M.H. (2022). Simulating in vitro the bone healing potential of a degradable and tailored multifunctional Mg-based alloy platform. Bioengineering-Basel.

[32] Aboutalebianaraki N., Zeblisky P., Sarker M.D., Jeyaranjan A., Sakthivel T.S., Fu Y., Lucchi J., Baudelet M., Seal S., Kean T.J., Razavi M. (2023). An osteogenic magnesium alloy with improved corrosion resistance, antibacterial, and mechanical properties for orthopedic applications. J. Biomed. Mater. Res..

[33] Zhang D., Peng F., Tan J., Zhang Y., Wang F., Xie J., Xu R., Du H., Qian S., Qiao Y. (2022). Self-assembled ferric oxyhydroxide nanosheet on PEO-coated magnesium alloy with photocatalytic/photothermal antibacterial and enhanced osteogenesis activities. Chem. Eng. J..

[34] Moreno J., Merlo J.L., Renno A.C., Canizo J., Buchelly F.J., Pastore J.I., Katunar M.R., Cere S. (2023). *In vitro* characterization of anodized magnesium alloy as a potential biodegradable material for biomedical applications. Electrochim. Acta.

[35] Kopp A., Fischer H., Soares A.P., Schmidt-Bleek K., Leber C., Kreiker H., Duda G., Kroeger N., van Gaalen K., Hanken H. (2023). Long-term *in vivo* observations show biocompatibility and performance of ZX00 magnesium screws surface-modified by plasma-electrolytic oxidation in Gottingen miniature pigs. Acta Biomater..

[36] Carnovali M., Mariotti M., Banfi G. (2021). Molecular hydrogen enhances osteogenesis in *Danio rerio* embryos. J. Fish. Biol..

[37] Kuhlmann J., Bartsch I., Willbold E., Schuchardt S., Holz O., Hort N., Hoeche D., Heineman W.R., Witte F. (2013). Fast escape of hydrogen from gas cavities around corroding magnesium implants. Acta Biomater..

[38] Hamushan M., Cai W., Zhang Y., Ren Z., Du J., Zhang S., Zhao C., Cheng P., Zhang X., Shen H., Han P. (2021). High-purity magnesium pin enhances bone consolidation in distraction osteogenesis via regulating Ptch protein activating Hedgehog-alternative Wnt signaling. Bioact. Mater..

[39] Sui B., Lu H., Liu X., Sun J. (2023). High-purity Mg and Mg-1Ca alloys: comparative assessment of the merits regarding degradation, osteogenesis, and biosafety for orthopedic applications. J. Mater. Sci. Technol..

[40] Ouyang J., Hong X., Gao Y. (2021). Retardation and self-repair of erosion pits by a two-stage barrier on bioactive-glass/layered double hydroxide coating of biomedical magnesium alloys. Surf. Coating. Technol..

[41] Pei Han PC., Zhang Shaoxiang, Zhao Changli, Ni Jiahua, Zhang Yuanzhuang, Zhong Wanrun, Hou Peng, Zhang Xiaonong, Zheng Yufeng (2015). Yimin Chai **in vitro and in vivo studies on the degradation of high-purity Mg (99.99wt.%) screw with femoral intracondylar fractured rabbit model**. Biomaterials.

[42] Bakhsheshi-Rad H.R., Idris M.H., Abdul-Kadir M.R., Ourdjini A., Medraj M., Daroonparvar M., Hamzah E. (2014). Mechanical and bio-corrosion properties of quaternary Mg-Ca-Mn-Zn alloys compared with binary Mg-Ca alloys. Mater. Des..

[43] Dong J., Lin P., Putra N.E., Tumer N., Leeflang M.A., Huan Z., Fratila-Apachitei L.E., Chang J., Zadpoor A.A., Zhou J. (2022). Extrusion-based additive manufacturing of Mg-Zn/bioceramic composite scaffolds. Acta Biomater..

[44] Kumar K.K.A., Srinivasan A., Pillai U.T.S., Pai B.C., Chakraborty M. (2022). Microstructure and mechanical property correlation of Mg-Si alloys. Silicon.

[45] Zhang E., Yin D., Xu L., Yang L., Yang K. (2009). Microstructure, mechanical and corrosion properties and biocompatibility of Mg-Zn-Mn alloys for biomedical application. Materials Science & Engineering C-Biomimetic and Supramolecular Systems.

[46] Hu Y., Dong D., Wang X., Chen H., Qiao Y. (2021). Synthesis and properties of Mg-Mn-Zn alloys for medical applications. Materials.

[47] Chandran V., Kunjan C., Veerapandian V., Kannan R. (2023). Mechanical, corrosion and biological behavior of centrifugal casting processed Mg-2Zn-1Mn alloy reinforced with β Tricalciumphosphate (βTCP) for orthopaedic applications. J. Mech. Behav. Biomed. Mater..

[48] Istrate B., Munteanu C., Matei M.N., Oprisan B., Moisei M., Earar K. (2016). International Conference on Modern Technologies in Industrial Engineering IV (ModTech).

[49] Li Y., Wen C., Mushahary D., Sravanthi R., Harishankar N., Pande G., Hodgson P. (2012). Mg-Zr-Sr alloys as biodegradable implant materials. Acta Biomater..

[50] Jin S., Zhang D., Lu X., Zhang Y., Tan L., Liu Y., Wang Q. (2020). Mechanical properties, biodegradability and cytocompatibility of biodegradable Mg-Zn-Zr-Nd/Y alloys. J. Mater. Sci. Technol..

[51] Chou D.-T., Hong D., Saha P., Ferrero J., Lee B., Tan Z., Dong Z., Kumta P.N. (2013). In vitro and in vivo corrosion, cytocompatibility and mechanical properties of biodegradable Mg-Y-Ca-Zr alloys as implant materials. Acta Biomater..

[52] Liu H., Sun C., Wang C., Li Y., Bai J., Xue F., Ma A., Jiang J. (2020). Improving toughness of a Mg_2_Ca-containing Mg-Al-Ca-Mn alloy via refinement and uniform dispersion of Mg_2_Ca particles. J. Mater. Sci. Technol..

[53] Asdi M.H., Khan M.U.A., Shafique A., Hussain J., Bashir S., Awan S., Shah S.A. (2023). Morphological, microstructural, mechanical, and electrochemical optimization of a novel Mg-2Ca-1Mn-1 Sr alloy by P ion implantation for orthopedic implants. Mater. Today Commun..

[54] Huang C.Y., Li D., Song J.L., Chen K., Wang X.L., Zhao F., Gu X.N., Xie X.H., Fan Y.B. (2023). Dual-functional coatings on magnesium alloys: enhancing corrosion behavior under stress and osteogenic effect in osteoporotic rats. Appl. Mater. Today.

[55] Ali A., Ikram F., Iqbal F., Fatima H., Mehmood A., Kolawole M.Y., Chaudhry A.A., Siddiqi S.A., Rehman I.U. (2021). Improving the in vitro degradation, mechanical and biological properties of AZ91-3Ca Mg alloy via hydrothermal calcium phosphate coatings. Frontiers in Materials.

[56] Wang W., Jia G., Wang Q., Huang H., Li X., Zeng H., Ding W., Witte F., Zhang C., Jia W., Yuan G. (2020). The in vitro and in vivo biological effects and osteogenic activity of novel biodegradable porous Mg alloy scaffolds. Mater. Des..

[57] Gusieva K.D.C.H.J., Scully J.R., Birbilis N. (2015). Corrosion of magnesium alloys: the role of alloying. Int. Mater. Rev..

[58] Zan R., Wang H., Cai W., Ni J., Luthringer-Feyerabend B.J.C., Wang W., Peng H., Ji W., Yan J., Xia J. (2022). Controlled release of hydrogen by implantation of magnesium induces P53-mediated tumor cells apoptosis. Bioact. Mater..

[59] Melia M.A., Florian D.C., Steuer F.W., Briglia B.F., Purzycki M.K., Scully J.R., Fitz-Gerald J.M. (2017). Investigation of critical processing parameters for laser surface processing of AZ31B-H24. Surf. Coating. Technol..

[60] Sahu M.R., Kumar T.S.S., Chakkingal U. (2021). Tailoring the properties of biodegradable Mg-Ca alloy by groove pressing technique. Trans. Indian Inst. Met..

[61] Yan Y., Cao H., Kang Y., Yu K., Xiao T., Luo J., Deng Y., Fang H., Xiong H., Dai Y. (2017). Effects of Zn concentration and heat treatment on the microstructure, mechanical properties and corrosion behavior of as-extruded Mg-Zn alloys produced by powder metallurgy. J. Alloys Compd..

[62] Cai S., Lei T., Li N., Feng F. (2012). Effects of Zn on microstructure, mechanical properties and corrosion behavior of Mg-Zn alloys. Mater. Sci. Eng., C.

[63] Hernandez L., Ramon-Sierra J., Soria-Castro M., Bacelis A., Rodriguez-Gattorno G., Ortiz-Vazquez E., Acosta G. (2023). Assessment of Mg(OH)2/TiO2 coating in the Mg-Ca-Zn alloy for improved corrosion resistance and antibacterial performance. J. Magnesium Alloys.

[64] Tsai M.-H., Yang C.-M., Chen Y.-H., Chen I.-G., Lin C.-F., Tseng C.-C. (2023). Hot pressing of DCPD-coated Mg-Zn-Ca powder: corrosion behavior observed using liquid cell transmission electron microscopy. Mater. Des..

[65] Yunpeng Hu, Xg, Yang Qiao, Wang Xiangyu (2022). Qichao Lin 2: **preparation of medical Mg–Zn alloys and the effect of different zinc contents on the alloy**. J. Mater. Sci. Mater. Med..

[66] Gu X.N., Xie X.H., Li N., Zheng Y.F., Qin L. (2012). In vitro and in vivo studies on a Mg-Sr binary alloy system developed as a new kind of biodegradable metal. Acta Biomater..

[67] Li Q., Xie B., Liu X., Liang W., Zhang X., Wang Y. (2022). Effects of Mg-Nd-Gd-Sr alloy on bone marrow mesenchymal stem cell function derived from SD rats. Eur. J. Inflamm..

[68] Xie J., Zhang Z., Liu S., Zhang J., Wang J., He Y., Lu L., Jiao Y., Wu R. (2023). Designing new low alloyed Mg-RE alloys with high strength and ductility via high-speed extrusion. Int. J. Miner. Metall. Mater..

[69] Chen K., Zhao Y., Liu C., Li Q., Bai Y., Li P., Wang C., Gu X., Fan Y. (2022). Novel Mg-Ca-La alloys for guided bone regeneration: mechanical performance, stress corrosion behavior and biocompatibility. Mater. Today Commun..

[70] Willbold E., Gu X., Albert D., Kalla K., Bobe K., Brauneis M., Janning C., Nellesen J., Czayka W., Tillmann W. (2015). Effect of the addition of low rare earth elements (lanthanum, neodymium, cerium) on the biodegradation and biocompatibility of magnesium. Acta Biomater..

[71] Chen L., Yan Z., Qiu T., Zhu J., Liu G., Han J., Guo C. (2023). Long-term temporospatial complementary relationship between degradation and bone regeneration of Mg-Al alloy. ACS Appl. Bio Mater..

[72] Zhang J., Qiao H., Li Q., Zhu P., Han C. (2024). Investigation of the microstructure, mechanical properties, in vitro degradation behavior, and biocompatibility of newly developed Zn-0.6%Li alloy. Adv. Eng. Mater..

[73] Zohrevand M., Alizadeh R., Mahmudi R. (2023). Using different strategies to improve properties of the biodegradable Mg-4Li-4Zn alloy. Journal of Materials Research and Technology-Jmr&T.

[74] Alp A., Rollas T., Eroglu E.O., Yildiz M.I., Yagcioglu A.E.A., Ulug B.D. (2024). Catatonia due to lithium neurotoxicity: a case report. Türk Psikiyatri Derg..

[75] Willumeit-Römer EPSNDKBZ-PDTBWFFTER (2021). Pore characterization of PM Mg–0.6Ca alloy and its degradation behavior under physiological conditions. J. Magnesium Alloys.

[76] Sahu M.R., Kumar T.S.S., Chakkingal U. (2022). A review on recent advancements in biodegradable Mg-Ca alloys. J. Magnesium Alloys.

[77] Nicholas T.K.N., Birbilis Jemimah, Walker Tim, Woodfield George J., Dias, Mark P. (2020). Staiger: **in-vitro dissolution of magnesium-calcium binary alloys: clarifying the unique role of calcium additions in bioresorbable magnesium implant alloys**. J. Biomed. Mater. Res. B Appl. Biomater..

[78] Wang Y., Tie D., Guan R., Wang N., Shang Y., Cui T., Li J. (2018). Microstructures, mechanical properties, and degradation behaviors of heat-treated Mg-Sr alloys as potential biodegradable implant materials. J. Mech. Behav. Biomed. Mater..

[79] Garimella A., Ramya M., Ghosh S.B., Bandyopadhyay-Ghosh S., Agrawal A.K. (2023). Bioactive fluorcanasite reinforced magnesium alloy-based porous bio-nanocomposite scaffolds with tunable mechanical properties. J. Biomed. Mater. Res. B Appl. Biomater..

[80] Wang Q., Xu L., Willumeit-Roemer R., Luthringer-Feyerabend B.J.C. (2021). Macrophage-derived oncostatin M/bone morphogenetic protein 6 in response to Mg-based materials influences pro-osteogenic activity of human umbilical cord perivascular cells. Acta Biomater..

[81] Tang H., Wang F., Li D., Gu X., Fan Y. (2020). Mechanical properties, degradation behaviors and biocompatibility of micro-alloyed Mg-Sr-RE alloys for stent applications. Mater. Lett..

[82] Wei X., Ma S., Meng J., Qing H., Zhao Q. (2023). *In vitro* evaluation of a novel Mg-Sn-Ge ternary alloy for orthopedic applications. J. Alloys Compd..

[83] Li K., Yu Y., Lu Q., Li Y., Yan Q., Lan X., Li L., Chen B., Song M. (2023). Microstructure and mechanical behavior of quaternary eutectic alpha+theta+Q+Si clusters in as-cast Al-Mg-Si-Cu alloys. Materials.

[84] Liu D., Yang D., Li X., Hu S. (2019). Mechanical properties, corrosion resistance and biocompatibilities of degradable Mg-RE alloys: a review. Journal of Materials Research and Technology-Jmr&T.

[85] Xue K., Tan P.-H., Zhao Z.-H., Cui L.-Y., Kannan M.B., Li S.-Q., Liu C.-B., Zou Y.-H., Zhang F., Chen Z.-Y., Zeng R.-C. (2023). In vitro degradation and multi-antibacterial mechanisms of beta-cyclodextrin@curcumin embodied Mg(OH)(2)/MAO coating on AZ31 magnesium alloy. J. Mater. Sci. Technol..

[86] Wang J., Deng S., Meng M., Tu W., Ou J. (2023). Improved corrosion resistance and biocompatibility of AZ31 alloy by acid pickling pretreatment and (H plus) hydroxyapatite/chitosan composite coating. Surf. Coating. Technol..

[87] Zhao G., Wang S., Wang G., Zhang B., Huang H., Yao Y. (2023). Enhancing bone formation using absorbable AZ31B magnesium alloy membranes during distraction osteogenesis: a new material study. Heliyon.

[88] Li J., Li J., He N., Fu Q., Feng M., Li Q., Wang Q., Liu X., Xiao S., Jin W. (2022). In situ growth of Ca-Zn-P coatings on the Zn-pretreated WE43 Mg alloy to mitigate corrosion and enhance cytocompatibility. Colloids Surf. B Biointerfaces.

[89] Witecka A., Valet S., Basista M., Boccaccini A.R. (2021). Electrophoretically deposited high molecular weight chitosan/bioactive glass composite coatings on WE43 magnesium alloy. Surf. Coating. Technol..

[90] Zhao Y., He P., Yao J., Li M., Wang B., Han L., Huang Z., Guo C., Bai J., Xue F. (2023). pH/NIR-responsive and self-healing coatings with bacteria killing, osteogenesis, and angiogenesis performances on magnesium alloy. Biomaterials.

[91] Jiang P., Hou R., Chen T., Bai L., Li J., Zhu S., Wang L., Willumeit-Roemer R., Guan S. (2023). Enhanced degradation performance and promoted bone regeneration of novel CaCO3-based hybrid coatings on magnesium alloy as bioresorbable orthopedic implants. Chem. Eng. J..

[92] Mao G., Jin X., Sun J., Han X., Zeng M., Qiu Y., Bian W. (2021). Microalloying design of biodegradable Mg-2Zn-0.05Ca promises improved bone-implant applications. ACS Biomater. Sci. Eng..

[93] Zhao K., Chen Y., Yu F., Jian W., Zheng M., Zeng H. (2022). A biodegradable magnesium alloy sample induced rat osteochondral defect repair through Wnt/beta-catenin signaling pathway. Advances in Nano Research.

[94] Yang H., Zhang F., Sun S., Li H., Li L., Xu H., Wang J., Shao M., Li C., Wang H. (2023). Brushite-coated Mg-Nd-Zn-Zr alloy promotes the osteogenesis of vertebral laminae through IGF2/PI3K/AKT signaling pathway. Biomater. Adv..

[95] Xie K., Wang L., Guo Y., Zhao S., Yang Y., Dong D., Ding W., Dai K., Gong W., Yuan G., Hao Y. (2021). Effectiveness and safety of biodegradable Mg-Nd-Zn-Zr alloy screws for the treatment of medial malleolar fractures. Journal of Orthopaedic Translation.

[96] Bagheri A., Sedighi M., Shamsi M. (2023). Effect of PCL/HA nanocomposite coating on the degradation rate and mechanical integrity of Mg/HA biocomposites during exposure in SBF. Arabian J. Sci. Eng..

[97] Amukarimi S., Mobasherpour I., Abdollahi S., Milan P.B., Mozafari M. (2023). Synthesis and characterization of ciprofloxacin-loaded biodegradable magnesium implants for the prevention of implant-associated infections. Mater. Chem. Phys..

[98] Liu Y., Li H., Xu J., TerBush J., Li W., Setty M., Guan S., Nguyen T.D., Qin L., Zheng Y. (2021). Biodegradable metal-derived magnesium and sodium enhances bone regeneration by angiogenesis aided osteogenesis and regulated biological apatite formation. Chem. Eng. J..

[99] Zhang W., Zhao S., Mo X., Xian P., Tang S., Qian J., Shen G., Zhou C., Huang N., Zhang H., Wan G. (2021). Mg ions incorporated phytic acid (PA) and zoledronic acid (ZA) of metal-organic complex coating on biodegradable magnesium for orthopedic implants application. Surf. Coating. Technol..

[100] Zheng H., Li Z., Chen M., You C., Wang J. (2023). Enhanced corrosion resistance and biocompatibility of NaMgF3 coating on Mg-Zn-Ca alloy: an in vivo and in vitro study. Adv. Eng. Mater..

[101] Li W., Tian A., Li T., Zhao Y., Chen M. (2023).

[102] Zhou Y.-L., Li Y., Luo D.-M., Wen C., Hodgson P. (2013). Microstructures, mechanical properties and in vitro corrosion behaviour of biodegradable Mg-Zr-Ca alloys. J. Mater. Sci..

[103] Ding Y., Li Y., Lin J., Wen C. (2015). Effects of zirconium and strontium on the biocorrosion of Mg-Zr-Sr alloys for biodegradable implant applications. J. Mater. Chem. B.

[104] Yuan B., Chen H., Zhao R., Deng X., Chen G., Yang X., Xiao Z., Aurora A., Iulia B.A., Zhang K. (2022). Construction of a magnesium hydroxide/graphene oxide/hydroxyapatite composite coating on Mg-Ca-Zn-Ag alloy to inhibit bacterial infection and promote bone regeneration. Bioact. Mater..

[105] Zhang Z.-Q., Wang L., Zeng M.-Q., Zeng R.-C., Kannan M.B., Lin C.-G., Zheng Y.-F. (2020). Biodegradation behavior of micro-arc oxidation coating on magnesium alloy-from a protein perspective. Bioact. Mater..

[106] Saranya K., Kalaiyarasan M., Agilan P., Rajendran N. (2022). Biofunctionalization of Mg implants with gadolinium coating for bone regeneration. Surf. Interfaces.

[107] Li H., Qin Z., Ouyang Y., Zheng B., Wei H., Ou J., Shen C. (2022). Hydroxyapatite/chitosan-metformin composite coating enhances the biocompatibility and osteogenic activity of AZ31 magnesium alloy. J. Alloys Compd..

[108] Peng F., Cheng S., Zhang R., Li M., Zhou J., Wang D., Zhang Y. (2021). Zn-contained mussel-inspired film on Mg alloy for inhibiting bacterial infection and promoting bone regeneration. Regenerative Biomaterials.

[109] Negrescu A.-M., Necula M.-G., Gebaur A., Golgovici F., Nica C., Curti F., Iovu H., Costache M., Cimpean A. (2021). In vitro macrophage immunomodulation by poly(epsilon-caprolactone) based-coated AZ31 Mg alloy. Int. J. Mol. Sci..

[110] Lin Y., Yang Y., Zhao Y., Gao F., Guo X., Yang M., Hong Q., Yang Z., Dai J., Pan C. (2021). Incorporation of heparin/BMP2 complex on GOCS-modified magnesium alloy to synergistically improve corrosion resistance, anticoagulation, and osteogenesis. J. Mater. Sci. Mater. Med..

[111] Liu Y., Cheng X., Wang X., Sun Q., Wang C., Di P., Lin Y. (2021). Micro-arc oxidation-assisted sol-gel preparation of calcium metaphosphate coatings on magnesium alloys for bone repair. Mater. Sci. Eng., C.

[112] Gao J., Su Y., Qin Y.-X. (2021). Calcium phosphate coatings enhance biocompatibility and degradation resistance of magnesium alloy: correlating in vitro and in vivo studies. Bioact. Mater..

[113] Khalili M.A., Tamjid E. (2021). Controlled biodegradation of magnesium alloy in physiological environment by metal organic framework nanocomposite coatings. Sci. Rep..

[114] Yang L., Shi X., Tian X., Wang H., Qi L. (2022). Microstructure and corrosion behavior of ZrO_2_ coated carbon fiber reinforced magnesium matrix composites sprayed with different powder characteristics. Ceram. Int..

[115] Wan X., Fang S., Xu S., Yu L., Zhou J., Qian S., Huang F., Ma C. (2024). Enhanced anti-corrosion and biological performance of plasma-sprayed Nb/ZrO_2_/HA coatings on ZK60 Mg alloy. Coatings.

[116] Rakoch A.G., Monakhova E.P., Khabibullina Z.V., Serdechnova M., Blawert C., Zheludkevich M.L., Gladkova A.A. (2020). Plasma electrolytic oxidation of AZ31 and AZ91 magnesium alloys: comparison of coatings formation mechanism. J. Magnesium Alloys.

[117] Liu B., Liu J., Wang C., Wang Z., Min S., Wang C., Zheng Y., Wen P., Tian Y. (2024). High temperature oxidation treated 3D printed anatomical WE43 alloy scaffolds for repairing periarticular bone defects: *in vitro* and *in vivo* studies. Bioact. Mater..

[118] Lin Z., Wang T., Yu X., Sun X., Yang H. (2021). Functionalization treatment of micro-arc oxidation coatings on magnesium alloys: a review. J. Alloys Compd..

[119] Vakili-Azghandi M., Fattah-Alhosseini A. (2017). Effects of duty cycle, current frequency, and current density on corrosion behavior of the plasma electrolytic oxidation coatings on 6061 Al alloy in artificial seawater. Metall. Mater. Trans. A.

[120] Li M., Yao M., Wang W., Wan P., Chu X., Zheng Y., Yang K., Zhang Y. (2021). Nitrogen-containing bisphosphonate-loaded micro-arc oxidation coating for biodegradable magnesium alloy pellets inhibits osteosarcoma through targeting of the mevalonate pathway. Acta Biomater..

[121] Luo H., Cai Q. (2011). High-temperature oxidaton performance of Micro-Arc film on AZ91D mgnesium alloy. Rare Met. Mater. Eng..

[122] Liu T., Li Y., Zhang Y., Zhao M., Wen Z., Zhang L. (2021). A biodegradable, mechanically tunable micro-arc oxidation AZ91D-based composite implant with calcium phosphate/chitosan coating promotes long-term bone tissue regeneration. Biotechnol. J..

[123] Park J.-E., Jang Y.-S., Seo J.-M., Lee M.-H. (2023). Facilitated osteogenesis of magnesium implant by coating of strontium incorporated calcium phosphate. Biointerphases.

[124] Li Q., Yan Y., Gao H. (2022). Improving the corrosion resistance and osteogenic differentiation of ZK60 magnesium alloys by hydroxyapatite/graphene/graphene oxide composite coating. Ceram. Int..

[125] Roshan S., Mohammadloo H.E., Sarabi A.A., Afshari M. (2022). Biocompatible hybrid chitosan/hydroxyapatite coating applied on the AZ31 Mg alloy substrate: in-vitro corrosion, surface and structure studies. Mater. Today Commun..

[126] Zhu Y., Zheng L., Liu W., Qin L., Ngai T. (2020). Poly(L-lactic acid) (PLLA)/MgSO_4_•7H_2_O composite coating on magnesium substrates for corrosion protection and cytocompatibility promotion. ACS Appl. Bio Mater..

[127] Shao Y., Zeng R.-C., Li S.-Q., Cui L.-Y., Zou Y.-H., Guan S.-K., Zheng Y.-F. (2020). Advance in antibacterial magnesium alloys and surface coatings on magnesium alloys: a review. Acta Metallurgica Sinica-English Letters.

[128] Zeng A., Wang Y., Li D., Guo J., Chen Q. (2021). Preparation and antibacterial properties of polycaprolactone/quaternized chitosan blends. Chin. J. Chem. Eng..

[129] Cui L-y, Xu J., Lu N., Zeng R-c, Zou Y-h, Li S-q, Zhang F. (2017). In vitro corrosion resistance and antibacterial properties of layer-by-layer assembled chitosan/poly-L-glutamic acid coating on AZ31 magnesium alloys. Trans. Nonferrous Metals Soc. China.

[130] Zhang D., Zhou J., Peng F., Tan J., Zhang X., Qian S., Qiao Y., Zhang Y., Liu X. (2022). Mg-Fe LDH sealed PEO coating on magnesium for biodegradation control, antibacteria and osteogenesis. J. Mater. Sci. Technol..

[131] Guo L., Wu W., Zhou Y., Zhang F., Zeng R., Zeng J. (2018). Layered double hydroxide coatings on magnesium alloys: a review. J. Mater. Sci. Technol..

[132] Cheng S., Shao H., Yin D., Zhou J., Jian L., Xie J., Zhang Y., Wang D., Peng F. (2023). Molecular mechanism underlying the action of a celastrol-loaded layered double hydroxide-coated magnesium alloy in osteosarcoma inhibition and bone regeneration. ACS Biomater. Sci. Eng..

[133] Naguib G.H., Abd El-Aziz G.S., Almehmadi A., Bayoumi A., Mira A.I., Hassan A.H., Hamed M.T. (2023). Evaluation of the time-dependent osteogenic activity of glycerol incorporated magnesium oxide nanoparticles in induced calvarial defects. Heliyon.

[134] Derakhshankhah H., Nekounam H., Izadi Z., Allahyari Z., Samari M., Feizi M., Samadian H. (2023). Fabrication of electroactive nanocomposite based on carbon nanofibers/magnesium oxide nanoparticles for bone tissue engineering. J. Drug Deliv. Sci. Technol..

[135] Li C., Zhang W., Wang R., Du X.-F., Jiang D., Liu B., Nie Y., Liao J., Chen Y., Liang X. (2022). Nanocomposite multifunctional hydrogel for suppressing osteosarcoma recurrence and enhancing bone regeneration. Chem. Eng. J..

[136] Li X., Dai B., Guo J., Zhu Y., Xu J., Xu S., Yao Z., Chang L., Li Y., He X. (2022). Biosynthesized bandages carrying magnesium oxide nanoparticles induce cortical bone formation by modulating endogenous periosteal cells. ACS Nano.

[137] Giron J., Kerstner E., Medeiros T., Oliveira L., Machado G.M., Malfatti C.F., Pranke P. (2021). Biomaterials for bone regeneration: an orthopedic and dentistry overview. Braz. J. Med. Biol. Res..

[138] Mahjoory M., Shahgholi M., Karimipour A. (2023). Investigation on the size and percentage effects of magnesium nanoparticles on thermophysical properties of reinforced calcium phosphate bone cement by molecular dynamic simulation. Heliyon.

[139] Chen R., Chen H.-B., Xue P.-P., Yang W.-G., Luo L.-Z., Tong M.-Q., Zhong B., Xu H.-L., Zhao Y.-Z., Yuan J.-D. (2021). HA/MgO nanocrystal-based hybrid hydrogel with high mechanical strength and osteoinductive potential for bone reconstruction in diabetic rats. J. Mater. Chem. B.

[140] Pan H., Gao H., Li Q., Lin Z., Feng Q., Yu C., Zhang X., Dong H., Chen D., Cao X. (2020). Engineered macroporous hydrogel scaffolds *via* pickering emulsions stabilized by MgO nanoparticles promote bone regeneration. J. Mater. Chem. B.

[141] Yang S., Liang L., Liu L., Yin Y., Liu Y., Lei G., Zhou K., Huang Q., Wu H. (2021). Using MgO nanoparticles as a potential platform to precisely load and steadily release Ag ions for enhanced osteogenesis and bacterial killing. Mater. Sci. Eng., C.

[142] Tang H., Zhang Y., Yang T., Wang C., Zhu Y., Qiu L., Liu J., Song Y., Zhou L., Zhang J. (2023). Cholesterol modulates the physiological response to nanoparticles by changing the composition of protein corona. Nat. Nanotechnol..

[143] Sabourian P., Yazdani G., Ashraf S.S., Frounchi M., Mashayekhan S., Kiani S., Kakkar A. (2020). Effect of physico-chemical properties of nanoparticles on their intracellular uptake. Int. J. Mol. Sci..

[144] Lu J., Sun M., Zhang J., Yang X., Dong M., He H., Liu A., Yu M., Wang B., Wang H. (2023). Benidipine-loaded nanoflower-likemagnesium silicate improves bone regeneration. Bio-Design and Manufacturing.

[145] Mahjoory M., Shahgholi M., Karimipour A. (2022). The effects of initial temperature and pressure on the mechanical properties of reinforced calcium phosphate cement with magnesium nanoparticles: a molecular dynamics approach. Int. Commun. Heat Mass Tran..

[146] Li X., Dai B., Guo J., Zhu Y., Xu J., Xu S., Yao Z., Chang L., Li Y., He X. (2022). Biosynthesized bandages carrying magnesium oxide nanoparticles induce cortical bone formation by modulating endogenous periosteal cells. ACS Nano.

[147] Ge Y., Wang K., Li H., Tian Y., Wu Y., Lin Z., Lin Y., Wang Y., Zhang J., Tang B. (2021). An Mg-MOFs based multifunctional medicine for the treatment of osteoporotic pain. Mater. Sci. Eng., C.

[148] Ma J., Yu H., Zhang X., Xu Z., Hu H., Liu J., Ren P., Kong X., Chen J., Yang K. (2024). Dual-targeted metal ion network hydrogel scaffold for promoting the integrated repair of tendon-bone interfaces. ACS Appl. Mater. Interfaces.

[149] Lawson S., Siemers A., Kostlenick J., Al-Naddaf Q., Newport K., Rownaghi A.A., Rezaei F. (2021). Mixing Mg-MOF-74 with Zn-MOF -74: a facile pathway of controlling the pharmacokinetic release rate of curcumin. ACS Appl. Bio Mater..

[150] Cooper L., Hidalgo T., Gorman M., Lozano-Fernandez T., Simon-Vazquez R., Olivier C., Guillou N., Serre C., Martineau C., Taulelle F. (2015). A biocompatible porous Mg-gallate metal-organic framework as an antioxidant carrier. Chem. Commun..

[151] Wang W., Xiong Y., Zhao R., Li X., Jia W. (2022). A novel hierarchical biofunctionalized 3D-printed porous Ti6Al4V scaffold with enhanced osteoporotic osseointegration through osteoimmunomodulation. J. Nanobiotechnol..

[152] Liu W., Yan Z., Ma X., Geng T., Wu H., Li Z. (2018). Mg-MOF-74/MgF_2_ composite coating for improving the properties of magnesium alloy implants: hydrophilicity and corrosion resistance. Materials.

[153] Wang B., Chen H., Peng S., Li X., Liu X., Ren H., Yan Y., Zhang Q. (2023). Multifunctional magnesium-organic framework doped biodegradable bone cement for antibacterial growth, inflammatory regulation and osteogenic differentiation. J. Mater. Chem. B.

[154] Zheng Z., Chen Y., Guo B., Wang Y., Liu W., Sun J., Wang X. (2020). Magnesium-organic framework-based stimuli-responsive systems that optimize the bone microenvironment for enhanced bone regeneration. Chem. Eng. J..

[155] Zhou Q., Su X., Wu J., Zhang X., Su R., Sun Q., Ma L., He R. (2023). Additive manufacturing of bioceramic implants for restoration bone engineering: technologies, advances, and future perspectives. ACS Biomater. Sci. Eng..

[156] Wang L., Pang Y., Tang Y., Wang X., Zhang D., Zhang X., Yu Y., Yang X., Cai Q. (2023). A biomimetic piezoelectric scaffold with sustained Mg2+ release promotes neurogenic and angiogenic differentiation for enhanced bone regeneration. Bioact. Mater..

[157] Jang H.J., Kang M.S., Jang J., Lim D., Choi S.-W., Jung T.-G., Chun H.-J., Kim B., Han D.-W. (2024). Harnessing 3D printed highly porous Ti-6Al-4V scaffolds coated with graphene oxide to promote osteogenesis. Biomater. Sci..

[158] He F., Rao J., Zhou J., Fu W., Wang Y., Zhang Y., Zuo F., Shi H. (2023). Fabrication of 3D printed Ca3Mg3(PO4)4-based bioceramic scaffolds with tailorable high mechanical strength and osteostimulation effect. Colloids Surf. B Biointerfaces.

[159] Xia X., Huang J., Wei J., Jin S., Zou Q., Zuo Y., Li J., Li Y. (2022). Magnesium oxide regulates the degradation behaviors and improves the osteogenesis of poly(lactide-co-glycolide) composite scaffolds. Compos. Sci. Technol..

[160] Lei B., Gao X., Zhang R., Yi X., Zhou Q. (2022). *In situ* magnesium phosphate/polycaprolactone 3D-printed scaffold induce bone regeneration in rabbit maxillofacial bone defect model. Mater. Des..

[161] Tan S., Wang Y., Du Y., Xiao Y., Zhang S. (2021). Injectable bone cement with magnesium-containing microspheres enhances osteogenesis via anti-inflammatory immunoregulation. Bioact. Mater..

[162] Wang L., Li Y., Jiang S., Zhang Z., Zhao S., Song Y., Liu J., Tan F. (2023). Alginate hydrogels containing different concentrations of magnesium-containing poly(lactic-co-glycolic acid) microspheres for bone tissue engineering. Biomed. Mater..

[163] Liang W., Gao M., Lou J., Bai Y., Zhang J., Lu T., Sun X., Ye J., Li B., Sun L. (2020). Integrating silicon/zinc dual elements with PLGA microspheres in calcium phosphate cement scaffolds synergistically enhances bone regeneration. J. Mater. Chem. B.

[164] Hou W., Guo J., Liu J., Zhao Y., Wei W., Shu D., Dai H. (2022). Calcium-magnesium phosphate biphasic microspheres with nutrient microchannels promote angiogenesis and osteogenic differentiation. Mater. Des..

[165] Yang F., Xu C., Zhang W., Sun L., Feng G., Ning T., Wang W., Sun B., Li J., Niu X., Fan Y. (2023). Biodegradable magnesium incorporated microspheres enable immunomodulation and spatiotemporal drug release for the treatment of osteonecrosis of the femoral head. Composites Part B-Engineering.

[166] Liu X., Ma Y., Chen M., Ji J., Zhu Y., Zhu Q., Guo M., Zhang P. (2021). Ba/Mg co-doped hydroxyapatite/PLGA composites enhance X-ray imaging and bone defect regeneration. J. Mater. Chem. B.

[167] Lin Z., Wu J., Qiao W., Zhao Y., Wong K.H.M., Chu P.K., Bian L., Wu S., Zheng Y., Cheung K.M.C. (2018). Precisely controlled delivery of magnesium ions thru sponge-like monodisperse PLGA/nano-MgO-alginate core-shell microsphere device to enable *in*-*situ* bone regeneration. Biomaterials.

[168] Cai Z., Liu X., Hu M., Meng Y., Zhao J., Tan Y., Luo X., Wang C., Ma J., Sun Z. (2023). In situ enzymatic reaction generates magnesium-based mineralized microspheres with superior bioactivity for enhanced bone regeneration. Adv. Healthcare Mater..

[169] Zhao Z., Li G., Ruan H., Chen K., Cai Z., Lu G., Li R., Deng L., Cai M., Cui W. (2021). Capturing magnesium ions via microfluidic hydrogel microspheres for promoting cancellous bone regeneration. ACS Nano.

[170] Yuan Z., Wan Z., Wei P., Lu X., Mao J., Cai Q., Zhang X., Yang X. (2020). Dual-controlled release of icariin/Mg2+ from biodegradable microspheres and their synergistic upregulation effect on bone regeneration. Adv. Healthcare Mater..

[171] Chen B., Liang Y., Bai L., Xu M., Zhang J., Guo B., Yin Z. (2020). Sustained release of magnesium ions mediated by injectable self-healing adhesive hydrogel promotes fibrocartilaginous interface regeneration in the rabbit rotator cuff tear model. Chem. Eng. J..

[172] He W., Chen K., Gao W., Duan R., Li Z., Li B., Xia J., Zhao Y., Liu W., Zhou H. (2024). A sequential physical and chemical dual crosslinked multifunctional hydrogel with enhanced mechanical and osteogenic effects for vascularized bone tissue regeneration. Mater. Des..

[173] Chen X., Tan B., Wang S., Tang R., Bao Z., Chen G., Chen S., Tang W., Wang Z., Long C. (2021). Rationally designed protein cross-linked hydrogel for bone regeneration via synergistic release of magnesium and zinc ions. Biomaterials.

[174] Guyot C., Cerruti M., Lerouge S. (2021). Injectable, strong and bioadhesive catechol-chitosan hydrogels physically crosslinked using sodium bicarbonate. Mater. Sci. Eng., C.

[175] Luo M., Chen M., Bai J., Chen T., He S., Peng W., Wang J., Zhi W., Weng J. (2022). A bionic composite hydrogel with dual regulatory functions for the osteochondral repair. Colloids Surf. B Biointerfaces.

[176] Xiong A., He Y., Gao L., Li G., Liu S., Weng J., Wang D., Zeng H. (2021). The fabrication of a highly efficient hydrogel based on a functionalized double network loaded with magnesium ion and BMP2 for bone defect synergistic treatment. Mater. Sci. Eng., C.

[177] Li J., Ke H., Lei X., Zhang J., Wen Z., Xiao Z., Chen H., Yao J., Wang X., Wei Z. (2024). Controlled-release hydrogel loaded with magnesium-based nanoflowers synergize immunomodulation and cartilage regeneration in tendon-bone healing. Bioact. Mater..

[178] He Y., Yang W., Zhang C., Yang M., Yu Y., Zhao H., Guan F., Yao M. (2024). ROS/pH dual responsive PRP-loaded multifunctional chitosan hydrogels with controlled release of growth factors for skin wound healing. Int. J. Biol. Macromol..

[179] Ma L., Cheng S., Ji X., Zhou Y., Zhang Y., Li Q., Tan C., Peng F., Zhang Y., Huang W. (2020). Immobilizing magnesium ions on 3D printed porous tantalum scaffolds with polydopamine for improved vascularization and osteogenesis. Mater. Sci. Eng., C.

[180] Tian L., Tang N., Ngai T., Wu C., Ruan Y., Huang L., Qin L. (2019). Hybrid fracture fixation systems developed for orthopaedic applications: a general review. Journal of Orthopaedic Translation.

[181] Zu H., Zheng L., Huo M., Liu K., Dreyer C.H., Zhang Y., He X., Li Y., Zou L., Huang L. (2024). Tree-inspired magnesium hybrid column for preventing hip collapse in steroid- associated osteonecrosis in bipedal emus. Mater. Today.

[182] Ibrahim M., Yu X., Tan L., Yang K. (2020). Influence of strontium phosphate coating on the degradation of physical vapor deposition sprayed Mg coating on Ti6Al4V substrate to promote bone tissue healing. Frontiers in Materials.

[183] Cotrut C.M., Ungureanu E., Ionescu I.C., Zamfir R.I., Kiss A.E., Parau A.C., Vladescu A., Vranceanu D.M., Saceleanu A. (2022). Influence of magnesium content on the physico-chemical properties of hydroxyapatite electrochemically deposited on a nanostructured titanium surface. Coatings.

[184] Cao J., Liu X., Jiang X., Lian R., Du B., Rogachev A.V. (2021). Studies of magnesium-hydroxyapatite micro/nano film for drug sustained release. Appl. Surf. Sci..

[185] Tao Z.-S., Zhou W.-S., He X.-W., Liu W., Bai B.-L., Zhou Q., Huang Z.-L., Tu K-k, Li H., Sun T. (2016). A comparative study of zinc, magnesium, strontium-incorporated hydroxyapatite-coated titanium implants for osseointegration of osteopenic rats. Mater. Sci. Eng., C.

[186] Shi S., Fan W., Tao R., Xu H., Lu Y., Han F., Yang S., Zhou X., Zhou Z., Wan F. (2021). Natural biomineralization-inspired magnesium silicate composite coating upregulates osteogenesis, enabling strong anterior cruciate ligament graft-bone healing in vivo. ACS Biomater. Sci. Eng..

[187] Florea D.A., Grumezescu V., Birca A.C., Vasile B.S., Musat M., Chircov C., Stan M.S., Grumezescu A.M., Andronescu E., Chifiriuc M.C. (2022). Design, characterization, and antibacterial performance of MAPLE-deposited coatings of magnesium phosphate-containing silver nanoparticles in biocompatible concentrations. Int. J. Mol. Sci..

[188] Siahmard P., Najafabadi R.A., Meysami A., Meysami M., Isfahani T. (2023). Investigation of structural properties of forsterite coating on AZ91 magnesium alloy by sol-gel method. Results in Engineering.

[189] Wang C., Zhang B., Yu S., Zhang H., Zhou W., Luo R., Wang Y., Bian W., Mao G. (2023). Incorporation of Mg-phenolic networks as a protective coating for magnesium alloy to enhance corrosion resistance and osteogenesis *in vivo*. J. Magnesium Alloys.

[190] Wei X., Zhou W., Tang Z., Wu H., Liu Y., Dong H., Wang N., Huang H., Bao S., Shi L. (2023). Magnesium surface-activated 3D printed porous PEEK scaffolds for *in vivo* osseointegration by promoting angiogenesis and osteogenesis. Bioact. Mater..

[191] Huang J., Ma Y., Pang K., Ma X., Zheng Z., Xu D., Xiong K., Yu B., Liao L. (2023). Anisotropic microspheres-cryogel composites loaded with magnesium l-threonate promote osteogenesis, angiogenesis, and neurogenesis for repairing bone defects. Biomacromolecules.

[192] Barbagallo M., Veronese N., Dominguez L.J. (2023). Magnesium-an ion with multiple invaluable actions, often insufficiently supplied: from in vitro to clinical research. Nutrients.

[193] Choi S., Kim K.-J., Cheon S., Kim E.-M., Kim Y.-A., Park C., Kim K.K. (2020). Biochemical activity of magnesium ions on human osteoblast migration. Biochem. Biophys. Res. Commun..

[194] Cerqueira A., Garcia-Arnaez I., Romero-Gavilan F., Azkargorta M., Elortza F., Martin de Llanos J.J., Carda C., Gurruchaga M., Goni I., Suay J. (2022). Complex effects of Mg-biomaterials on the osteoblast cell machinery: a proteomic study. Biomater. Adv..

[195] Wang C.-X., Ma T., Wang M.-Y., Guo H.-Z., Ge X.-Y., Zhang Y., Lin Y. (2021). Facile distribution of an alkaline microenvironment improves human bone marrow mesenchymal stem cell osteogenesis on a titanium surface through the ITG/FAK/ALP pathway. Int. J. Implant Dent..

[196] Wang N., Yang S., Shi H., Song Y., Sun H., Wang Q., Tan L., Guo S. (2022). Magnesium alloys for orthopedic applications:A review on the mechanisms driving bone healing. J. Magnesium Alloys.

[197] Leem Y.-H., Lee K.-S., Kim J.-H., Seok H.-K., Chang J.-S., Lee D.-H. (2016). Magnesium ions facilitate integrin alpha 2-and alpha 3-mediated proliferation and enhance alkaline phosphatase expression and activity in hBMSCs. Journal of Tissue Engineering and Regenerative Medicine.

[198] Zreiqat H., Howlett C.R., Zannettino A., Evans P., Schulze-Tanzil G., Knabe C., Shakibaei M. (2002). Mechanisms of magnesium-stimulated adhesion of osteoblastic cells to commonly used orthopaedic implants. J. Biomed. Mater. Res..

[199] Nunes A.M., Minetti C.A.S.A., Remeta D.P., Baum J. (2018). Magnesium activates microsecond dynamics to regulate integrin-collagen recognition. Structure.

[200] Nie X., Sun X., Wang C., Yang J. (2020). Effect of magnesium ions/Type I collagen promote the biological behavior of osteoblasts and its mechanism. Regenerative Biomaterials.

[201] Yu L., Xia K., Gong C., Chen J., Li W., Zhao Y., Guo W., Dai H. (2020). An injectable bioactive magnesium phosphate cement incorporating carboxymethyl chitosan for bone regeneration. Int. J. Biol. Macromol..

[202] Qi H., Liu Y., Wu L., Ni S., Sun J., Xue J., Liu Q., Ni X., Fan W. (2020). MicroRNA-16, via FGF2 regulation of the ERK/MAPK pathway, is involved in the magnesium-promoted osteogenic differentiation of mesenchymal stem cells. Oxid. Med. Cell. Longev..

[203] Wang Y., Geng Z., Huang Y., Jia Z., Cui Z., Li Z., Wu S., Liang Y., Zhu S., Yang X., Lu W.W. (2018). Unraveling the osteogenesis of magnesium by the activity of osteoblasts *in vitro*. J. Mater. Chem. B.

[204] Wang Z., Liu Q., Liu C., Tan W., Tang M., Zhou X., Sun T., Deng Y. (2020). Mg^2+^ in β-TCP/Mg-Zn composite enhances the differentiation of human bone marrow stromal cells into osteoblasts through MAPK-regulated Runx2/Osx. J. Cell. Physiol..

[205] Sarian M.N., Iqbal N., Sotoudehbagha P., Razavi M., Ahmed Q.U., Sukotjo C., Hermawan H. (2022). Potential bioactive coating system for high-performance absorbable magnesium bone implants. Bioact. Mater..

[206] Hou P., Sun Y., Yang W., Wu H., Sun L., Xiu X., Xiu C., Zhang X., Zhang W. (2022). Magnesium promotes osteogenesis via increasing OPN expression and activating CaM/CaMKIV/CREB1 pathway. J. Biomed. Mater. Res. B Appl. Biomater..

[207] Xie Y., Bao Z., Wang Z., Du D., Chen G., Liu C., Wang H., Feng N., Xiao X., Wang S. (2023). Magnesium ascorbyl phosphate promotes bone formation via CaMKII signaling. J. Bone Miner. Res..

[208] Lin Z., Shen D., Zhou W., Zheng Y., Kong T., Liu X., Wu S., Chu P.K., Zhao Y., Wu J. (2021). Regulation of extracellular bioactive cations in bone tissue microenvironment induces favorable osteoimmune conditions to accelerate *in situ* bone regeneration. Bioact. Mater..

[209] Ding S., Zhang J., Tian Y., Huang B., Yuan Y., Liu C. (2016). Magnesium modification up-regulates the bioactivity of bone morphogenetic protein-2 upon calcium phosphate cement via enhanced BMP receptor recognition and Smad signaling pathway. Colloids Surf. B Biointerfaces.

[210] Hung C.-C., Chaya A., Liu K., Verdelis K., Sfeir C. (2019). The role of magnesium ions in bone regeneration involves the canonical Wnt signaling pathway. Acta Biomater..

[211] Yu L., Gao T., Li W., Yang J., Liu Y., Zhao Y., He P., Li X., Guo W., Fan Z., Dai H. (2023). Carboxymethyl chitosan-alginate enhances bone repair effects of magnesium phosphate bone cement by activating the FAK-Wnt pathway. Bioact. Mater..

[212] Zhu Y., Jia G., Yang Y., Weng J., Liu S., Zhang M., Zhang G., Qin H., Chen Y., Yang Q. (2023). Biomimetic porous magnesium alloy scaffolds promote the repair of osteoporotic bone defects in rats through activating the wnt/β-catenin signaling pathway. ACS Biomater. Sci. Eng..

[213] Xiaofeng Q.I.P.S., Coutavas Elias, Xiaochun L.I. (2018). Two Patched molecules engage distinct sites on Hedgehog yielding a signaling-competent complex. Science.

[214] Xin Gong H.Q., Cao Ping, Zhao X.I.N., Zhou Qiang, Jianlin L.E.I., Yan Nieng (2018). Structural basis for the recognition of sonic hedgehog by human Patched1. Science.

[215] He X., Li Y., Miao H., Xu J., Ong MT-y, Wang C., Zheng L., Wang J., Huang L., Zu H. (2024). High formability Mg-Zn-Gd wire facilitates ACL reconstruction via its swift degradation to accelerate intra-tunnel endochondral ossification. J. Magnesium Alloys.

[216] Liu J., Zeng H., Xiao P., Yang A., Situ X., Wang Y., Zhang X., Li W., Pan W., Wang Y. (2020). Sustained release of magnesium ions mediated by a dynamic mechanical hydrogel to enhance BMSC proliferation and differentiation. ACS Omega.

[217] Wu Q., Xu S., Wang F., He B., Wang X., Sun Y., Ning C., Dai K. (2021). Double-edged effects caused by magnesium ions and alkaline environment regulate bioactivities of magnesium-incorporated silicocarnotite *in vitro*. Regenerative Biomaterials.

[218] Zhang X., Zu H., Zhao D., Yang K., Tian S., Yu X., Lu F., Liu B., Yu X., Wang B. (2017). Ion channel functional protein kinase TRPM7 regulates Mg ions to promote the osteoinduction of human osteoblast via PI3K pathway: *in vitro* simulation of the bone-repairing effect of Mg-based alloy implant. Acta Biomater..

[219] Wang Z., Wang X., Tian Y., Pei J., Zhang J., Jiang C., Huang J., Pang Z., Cao Y., Wang X. (2020). Degradation and osteogenic induction of a SrHPO_4_-coated Mg-Nd-Zn-Zr alloy intramedullary nail in a rat femoral shaft fracture model. Biomaterials.

[220] Zheng L.-Z., Wang J.-L., Xu J.-K., Zhang X.-T., Liu B.-Y., Huang L., Zhang R., Zu H.-Y., He X., Mi J. (2020). Magnesium and vitamin C supplementation attenuates steroid-associated osteonecrosis in a rat model. Biomaterials.

[221] Sargenti A., Castiglioni S., Olivi E., Bianchi F., Cazzaniga A., Farruggia G., Cappadone C., Merolle L., Malucelli E., Ventura C. (2018). Magnesium deprivation potentiates human mesenchymal stem cell transcriptional remodeling. Int. J. Mol. Sci..

[222] Liu Q., Yu Y., Liu C., Liu Y., Yuan L., Wang Z., Yu A. (2023). Effect of La^3+^ and Mg^2+^ combined system on bioactivity and osteogenesis of bioinspired La- doped magnesium phosphate composites prepared utilizing the precursor method. Journal of Materials Research and Technology-Jmr&T.

[223] Li Y., Wang J., Yue J., Wang Y., Yang C., Cui Q. (2018). High magnesium prevents matrix vesicle-mediated mineralization in human bone marrow-derived mesenchymal stem cells via mitochondrial pathway and autophagy. Cell Biol. Int..

[224] Lin S., Yang G., Jiang F., Zhou M., Yin S., Tang Y., Tang T., Zhang Z., Zhang W., Jiang X. (2019). A magnesium-enriched 3D culture system that mimics the bone development microenvironment for vascularized bone regeneration. Adv. Sci..

[225] Yuan Z., Wei P., Huang Y., Zhang W., Chen F., Zhang X., Mao J., Chen D., Cai Q., Yang X. (2019). Injectable PLGA microspheres with tunable magnesium ion release for promoting bone regeneration. Acta Biomater..

[226] Wang J., Ma X.-Y., Feng Y.-F., Ma Z.-S., Ma T.-C., Zhang Y., Li X., Wang L., Lei W. (2017). Magnesium ions promote the biological behaviour of rat calvarial osteoblasts by activating the PI3K/Akt signalling pathway. Biol. Trace Elem. Res..

[227] Ricchiuto S., Palumbo R., Lami F., Gavioli F., Caselli L., Montanari M., Zappavigna V., Anesi A., Zanocco-Marani T., Grande A. (2023). The capacity of magnesium to induce osteoclast differentiation is greatly enhanced by the presence of zoledronate. Biology-Basel.

[228] Belluci M.M., de Molon R.S., Rossa C., Tetradis S., Giro G., Cerri P.S., Marcantonio E. (2020). Peres Orrico SR: **severe magnesium deficiency compromises systemic bone mineral density and aggravates inflammatory bone resorption**. JNB (J. Nutr. Biochem.).

[229] Mammoli F., Castiglioni S., Parenti S., Cappadone C., Farruggia G., Iotti S., Davalli P., Maier J.A.M., Grande A., Frassineti C. (2019). Magnesium is a key regulator of the balance between osteoclast and osteoblast differentiation in the presence of vitamin D_3_. Int. J. Mol. Sci..

[230] Liu Y., Wang W., Zeng Y., Zeng H. (2023). Transcriptome analysis of hydrogen inhibits osteoclastogenesis of mouse bone marrow mononuclear cells. Exp. Ther. Med..

[231] Hablas N.M.M., Keshk W.A.A. (2023). OPG/RANK/RANKL Axis in Egyptian children with acute lymphoblastic leukemia after maintenance therapy: relationship to bone mineral and vitamin D status. Journal of Pediatric Hematology Oncology.

[232] Shibutani T., Heersche J.N. (1993). Effect of medium pH on osteoclast activity and osteoclast formation in cultures of dispersed rabbit osteoclasts. J. Bone Miner. Res. : the official journal of the American Society for Bone and Mineral Research.

[233] Chen M., Hu Y., Hou Y., Li M., Tan L., Chen M., Geng W., Tao B., Jiang H., Luo Z., Cai K. (2022). Magnesium/gallium-layered nanosheets on titanium implants mediate osteogenic differentiation of MSCs and osseointegration under osteoporotic condition. Chem. Eng. J..

[234] Liu Y., Wang D.-L., Huang Y.-C., Wang T.-B., Zeng H. (2020). Hydrogen inhibits the osteoclastogenesis of mouse bone marrow mononuclear cells. Mater. Sci. Eng., C.

[235] Chen Z., Klein T., Murray R.Z., Crawford R., Chang J., Wu C., Xiao Y. (2016). Osteoimmunomodulation for the development of advanced bone biomaterials. Mater. Today.

[236] Schumacher S., Roth I., Stahl J., Baeumer W., Kietzmann M. (2014). Biodegradation of metallic magnesium elicits an inflammatory response in primary nasal epithelial cells. Acta Biomater..

[237] Liang L., Song D., Wu K., Ouyang Z., Huang Q., Lei G., Zhou K., Xiao J., Wu H. (2022). Sequential activation of M1 and M2 phenotypes in macrophages by Mg degradation from Ti-Mg alloy for enhanced osteogenesis. Biomater. Res..

[238] Jeremy I., Pearl aTM., Irani a Afraaz R., Huang a Zhinong, Robinson a William H., Smith b Robert L., Goodmana Stuart B. (2011). ∗: **role of the Toll-like receptor pathway in the recognition of orthopedic implant wear-debris particles**. HHS Author Manuscripts.

[239] Zhu Y., Zhao S., Cheng L., Lin Z., Zeng M., Ruan Z., Sun B., Luo Z., Tang Y., Long H. (2022). Mg^2+^-mediated autophagy-dependent polarization of macrophages mediates the osteogenesis of bone marrow stromal stem cells by interfering with macrophage-derived exosomes containing miR-381. J. Orthop. Res..

[240] Bessa-Goncalves M., Silva A.M., Bras J.P., Helmholz H., Luthringer-Feyerabend B.J.C., Willumeit-Roemer R., Barbosa M.A., Santos S.G. (2020). Fibrinogen and magnesium combination biomaterials modulate macrophage phenotype, NF-kB signaling and crosstalk with mesenchymal stem/stromal cells. Acta Biomater..

[241] Rios F.J., Zou Z.-G., Harvey A.P., Harvey K.Y., Nosalski R., Anyfanti P., Camargo L.L., Lacchini S., Ryazanov A.G., Ryazanova L. (2020). Chanzyme TRPM7 protects against cardiovascular inflammation and fibrosis. Cardiovasc. Res..

[242] Chen L., Zhu J., Ge N., Liu Y., Yan Z., Liu G., Li Y., Wang Y., Wu G., Qiu T. (2025). A biodegradable magnesium alloy promotes subperiosteal osteogenesis via interleukin-10-dependent macrophage immunomodulation. Biomaterials.

[243] Xie K., Wang N.Q., Guo Y., Zhao S., Tan J., Wang L., Li G.Y., Wu J.X., Yang Y.Z., Xu W.Y. (2022). Additively manufactured biodegradable porous magnesium implants for elimination of implant-related infections: an in vitro and in vivo study. Bioact. Mater..

[244] Sun L., Li X., Xu M., Yang F., Wang W., Niu X. (2020). *In vitro* immunomodulation of magnesium on monocytic cell toward anti-inflammatory macrophages. Regenerative Biomaterials.

[245] Ozen M., Xie H., Shin N., Al Yousif G., Clemens J., McLane M.W., Lei J., Burd I. (2020). Magnesium sulfate inhibits inflammation through P2X7 receptors in human umbilical vein endothelial cells. Pediatr. Res..

[246] Maier J.A.M., Locatelli L., Fedele G., Cazzaniga A., Mazur A. (2023). Magnesium and the brain: a focus on neuroinflammation and neurodegeneration. Int. J. Mol. Sci..

[247] Qiao W., Pan D., Zheng Y., Wu S., Liu X., Chen Z., Wan M., Feng S., Cheung K.M.C., Yeung K.W.K., Cao X. (2022). Divalent metal cations stimulate skeleton interoception for new bone formation in mouse injury models. Nat. Commun..

[248] Xing Y., Zhong X., Chen S., Wu S., Chen K., Li X., Su M., Liu X., Zhong J., Chen Z. (2023). Optimized osteogenesis of porcine bone-derived xenograft through surface coating of magnesium-doped nanohydroxyapatite. Biomed. Mater..

[249] Diomede F., Marconi G.D., Fonticoli L., Pizzicanella J., Merciaro I., Bramanti P., Mazzon E., Trubiani O. (2020). Functional relationship between osteogenesis and angiogenesis in tissue regeneration. Int. J. Mol. Sci..

[250] Shi Z., Huang G., Li Z., Lou Z., Gong Z., Wang X., Li C., Wang B. (2023). A PLA-tPU based magnesium ion incorporated CSH/nHA bioactive porous composite scaffold for critical bone defect repair. Materials Advances.

[251] Luo R., Huang Y., Yuan X., Yuan Z., Zhang L., Han J., Zhao Y., Cai Q. (2021). Controlled co-delivery system of magnesium and lanthanum ions for vascularized bone regeneration. Biomed. Mater..

[252] Coelho C.C., Padrao T., Costa L., Pinto M.T., Costa P.C., Domingues V.F., Quadros P.A., Monteiro F.J., Sousa S.R. (2020). The antibacterial and angiogenic effect of magnesium oxide in a hydroxyapatite bone substitute. Sci. Rep..

[253] Sreenivasamurthy S.A., Akhter F.F., Akhter A., Su Y., Zhu D. (2022). Cellular mechanisms of biodegradable zinc and magnesium materials on promoting angiogenesis. Biomater. Adv..

[254] Gao P., Fan B., Yu X., Liu W., Wu J., Shi L., Yang D., Tan L., Wan P., Hao Y. (2020). Biofunctional magnesium coated Ti6Al4V scaffold enhances osteogenesis and angiogenesis *in vitro* and *in vivo* for orthopedic application. Bioact. Mater..

[255] Xie H., Cui Z., Wang L., Xia Z., Hu Y., Xian L., Li C., Xie L., Crane J., Wan M. (2014). PDGF-BB secreted by preosteoclasts induces angiogenesis during coupling with osteogenesis. Nat. Med..

[256] Liu W., Guo S., Tang Z., Wei X., Gao P., Wang N., Li X., Guo Z. (2020). Magnesium promotes bone formation and angiogenesis by enhancing MC3T3-E1 secretion of PDGF-BB. Biochem. Biophys. Res. Commun..

[257] Qin H., Weng J., Zhou B., Zhang W., Li G., Chen Y., Qi T., Zhu Y., Yu F., Zeng H. (2023). Magnesium ions promote in vitro rat bone marrow stromal cell angiogenesis through Notch signaling. Biol. Trace Elem. Res..

[258] Yoshizawa S., Brown A., Barchowsky A., Sfeir C. (2014). Magnesium ion stimulation of bone marrow stromal cells enhances osteogenic activity, simulating the effect of magnesium alloy degradation. Acta Biomater..

[259] Wei L., Du Z., Zhang C., Zhou Y., Zhu F., Chen Y., Zhao H., Zhang F., Dang P., Wang Y. (2023). Mg-CS/HA microscaffolds display excellent biodegradability and controlled release of Si and Mg bioactive ions to synergistically promote vascularized bone regeneration. Adv. Mater. Interfac..

[260] Xiong Y., Lin Z., Bu P., Yu T., Endo Y., Zhou W., Sun Y., Cao F., Dai G., Hu Y. (2023). A whole-course-repair system based on neurogenesis-angiogenesis crosstalk and macrophage reprogramming promotes diabetic wound healing. Adv. Mater..

[261] Li Y., Xu J., Mi J., He X., Pan Q., Zheng L., Zu H., Chen Z., Dai B., Li X. (2021). Biodegradable magnesium combined with distraction osteogenesis synergistically stimulates bone tissue regeneration via CGRP-FAK-VEGF signaling axis. Biomaterials.

[262] Fukuda T., Takeda S., Xu R., Ochi H., Sunamura S., Sato T., Shibata S., Yoshida Y., Gu Z., Kimura A. (2013). Sema3A regulates bone-mass accrual through sensory innervations. Nature.

[263] Li Q., Liu W., Hou W., Wu X., Wei W., Liu J., Hu Y., Dai H. (2023). Micropatterned photothermal double-layer periosteum with angiogenesis-neurogenesis coupling effect for bone regeneration. Materials Today Bio.

[264] Chen H., Hu B., Lv X., Zhu S., Zhen G., Wan M., Jain A., Gao B., Chai Y., Yang M. (2019). Prostaglandin E2 mediates sensory nerve regulation of bone homeostasis. Nat. Commun..

[265] Li Y., Hoffman M.D., Benoit D.S.W. (2021). Matrix metalloproteinase (MMP)-degradable tissue engineered periosteum coordinates allograft healing via early stage recruitment and support of host neurovasculature. Biomaterials.

[266] Zhang Z., Hao Z., Xian C., Fang Y., Cheng B., Wu J., Xia J. (2022). Neuro-bone tissue engineering: multiple potential translational strategies between nerve and bone. Acta Biomater..

[267] Zhao J., Zhang S., Duan L., Yao R., Yan Y., Wang T., Wang J., Zheng Z., Wang X., Li G. (2022). Preparation and mechanical optimization of a two-layer silk/magnesium wires braided porous artificial nerve guidance conduit. J. Biomed. Mater. Res..

[268] Hopkins T.M., Little K.J., Vennemeyer J.J., Triozzi J.L., Turgeon M.K., Heilman A.M., Minteer D., Marra K., Hom D.B., Pixley S.K. (2017). Short and long gap peripheral nerve repair with magnesium metal filaments. J. Biomed. Mater. Res..

[269] Yao Z., Yuan W., Xu J., Tong W., Mi J., Ho P.-C., Chow D.H.K., Li Y., Yao H., Li X. (2022). Magnesium-encapsulated injectable hydrogel and 3D-engineered polycaprolactone conduit facilitate peripheral nerve regeneration. Adv. Sci..

[270] Chang Y.-Y., Kao M.-C., Lin J.-A., Chen T.-Y., Cheng C.-F., Wong C.-S., Tzeng I.S., Huang C.-J. (2018). Effects of MgSO_4_ on inhibiting Nod-like receptor protein 3 inflammasome involve decreasing intracellular calcium. J. Surg. Res..

[271] Chen R., Hao Z., Chen X., Fu Q., Ma Y. (2020). Neuropeptide Y enhances proliferation and chondrogenic differentiation of ATDC5 cells. Neuropeptides.

[272] Yao Z., Chen Z., He X., Wei Y., Qian J., Zong Q., He S., Song L., Ma L., Lin S. (2024). Bioactive MgO/MgCO_3_/polycaprolactone multi-gradient fibers facilitate peripheral nerve regeneration by regulating schwann cell function and activating wingless/integrase-1 signaling. Advanced Fiber Materials.

[273] Zhang J., Zhang B., Zhang J., Lin W., Zhang S. (2021). Magnesium promotes the regeneration of the peripheral nerve. Front. Cell Dev. Biol..

[274] Zhang Y., Xu J., Ruan Y.C., Yu M.K., O'Laughlin M., Wise H., Chen D., Tian L., Shi D., Wang J. (2016). Implant-derived magnesium induces local neuronal production of CGRP to improve bone-fracture healing in rats. Nat. Med..

[275] Li C., Zhang W., Nie Y., Du X., Huang C., Li L., Long J., Wang X., Tong W., Qin L., Lai Y. (2023). Time-sequential and multi-functional 3D printed MgO_2_/PLGA scaffold developed as a novel biodegradable and bioactive bone substitute for challenging postsurgical osteosarcoma treatment. Adv. Mater..

[276] Shi H., Chen M. (2024). The brain-bone axis: unraveling the complex interplay between the central nervous system and skeletal metabolism. Eur. J. Med. Res..

[277] Miyashita T., Koda M., Kitajo K., Yamazaki M., Takahashi K., Kikuchi A., Yamashita T. (2009). Wnt-ryk signaling mediates axon growth inhibition and limits functional recovery after spinal cord injury. J. Neurotrauma.

[278] Li J., Zhou P., Wang L., Hou Y., Zhang X., Zhu S., Guan S. (2021). Investigation of Mg-*x*Li-Zn alloys for potential application of biodegradable bone implant materials. J. Mater. Sci. Mater. Med..

[279] Xie J., Cheng C-s, Zhu X.Y., Shen Y.H., Song L.B., Chen H., Chen Z., Liu L.M., Meng Z.Q. (2019). Magnesium transporter protein solute carrier family 41 member 1 suppresses human pancreatic ductal adenocarcinoma through magnesium-dependent Akt/mTOR inhibition and bax-associated mitochondrial apoptosis. Aging-Us.

[280] Zan R., Ji W., Qiao S., Wu H., Wang W., Ji T., Yang B., Zhang S., Luo C., Song Y. (2021). Biodegradable magnesium implants: a potential scaffold for bone tumor patients. Sci. China Mater..

[281] Zhang X., Lin Y., Gillies R.J. (2010). Tumor pH and its measurement. J. Nucl. Med..

[282] Li M., Ren L., Li L., He P., Lan G., Zhang Y., Yang K. (2014). Cytotoxic effect on osteosarcoma MG-63 cells by degradation of magnesium. J. Mater. Sci. Technol..

[283] Heiden M.G.V., Cantley L.C., Thompson C.B. (2009). Understanding the Warburg effect: the metabolic requirements of cell proliferation. Science.

[284] Jiang Y., Liu G., Zhang L., Cheng S., Luo C., Liao Y., Guo S. (2018). Therapeutic efficacy of hydrogen-rich saline alone and in combination with PI3K inhibitor in non-small cell lung cancer. Mol. Med. Rep..

[285] Ma N., Chen Y., Yang B. (2014). Magnesium metal-A potential biomaterial with antibone cancer properties. J. Biomed. Mater. Res..

[286] Meng X., Chen H., Wang G., Yu Y., Xie K. (2015). Hydrogen-rich saline attenuates chemotherapy-induced ovarian injury via regulation of oxidative stress. Exp. Ther. Med..

[287] Long J., Zhang W., Chen Y., Teng B., Liu B., Li H., Yao Z., Wang D., Li L., Yu X.-F. (2021). Multifunctional magnesium incorporated scaffolds by 3D-Printing for comprehensive postsurgical management of osteosarcoma. Biomaterials.

[288] Liu L., Peng F., Zhang D., Li M., Huang J., Liu X. (2022). A tightly bonded reduced graphene oxide coating on magnesium alloy with photothermal effect for tumor therapy. J. Magnesium Alloys.

[289] Ma L., Feng X., Liang H., Wang K., Song Y., Tan L., Wang B., Luo R., Liao Z., Li G. (2020). A novel photothermally controlled multifunctional scaffold for clinical treatment of osteosarcoma and tissue regeneration. Mater. Today.

[290] Ge J., Yang N., Yang Y., Yu H., Yang X., Wang Y., Wang T., Cheng S., Wang Y., Han Z. (2023). The combination of eddy thermal effect of biodegradable magnesium with immune checkpoint blockade shows enhanced efficacy against osteosarcoma. Bioact. Mater..

[291] Li C., Zhang W., Nie Y., Du X., Huang C., Li L., Long J., Wang X., Tong W., Qin L., Lai Y. (2024). Time-sequential and multi-functional 3D printed MgO_2_/PLGA scaffold developed as a novel biodegradable and bioactive bone substitute for challenging postsurgical osteosarcoma treatment. Adv. Mater..

[292] Ferreira C.A.M., Guerreiro S.F.C., Valente J.F.A., Patricio T.M.F., Alves N., Mateus A., Dias J.R. (2022). Advanced face mask filters based on PCL electrospun meshes dopped with antimicrobial MgO and CuO nanoparticles. Polymers.

[293] Xie K., Wang N., Guo Y., Zhao S., Tan J., Wang L., Li G., Wu J., Yang Y., Xu W. (2022). Additively manufactured biodegradable porous magnesium implants for elimination of implant-related infections: an *in vitro* and *in vivo* study. Bioact. Mater..

[294] Zhang X., Yin H., Xiao L., Li Z., Ma C., Xu W., Wang Y. (2022). Chitosan regulated electrochemistry for dense hydroxyapatite/MgO nanocomposite coating with antibiosis and osteogenesis on titanium alloy. Colloid and Interface Science Communications.

[295] Spiller F., Formiga R.O., da Silva Coimbra J.F., Alves-Filho J.C., Cunha T.M., Cunha F.Q. (2019). Targeting nitric oxide as a key modulator of sepsis, arthritis and pain. Nitric Oxide-Biology and Chemistry.

[296] Chen K., Ge W., Zhao L., Kong L., Yang H., Zhang X., Gu X., Zhu C., Fan Y. (2023). Endowing biodegradable Zinc implants with dual-function of antibacterial ability and osteogenic activity by micro-addition of Mg and Ag (≤ 0.1 wt.%). Acta Biomater..

[297] Jeena T., Geetha M.P., Suchetan P.A., Ronald N., Amrutha K. (2023). Influence of cobalt doping on chemical and green synthesized magnesium oxide nanoparticles for enhanced photocatalytic evaluation, adsorption studies, antimicrobial analysis and corrosion inhibition study. Inorg. Chem. Commun..

[298] Windhagen H., Radtke K., Weizbauer A., Diekmann J., Noll Y., Kreimeyer U., Schavan R., Stukenborg-Colsman C., Waizy H. (2013). Biodegradable magnesium-based screw clinically equivalent to titanium screw in hallux valgus surgery: short term results of the first prospective, randomized, controlled clinical pilot study. Biomed. Eng. Online.

[299] Zhao D., Huang S., Lu F., Wang B., Yang L., Qin L., Yang K., Li Y., Li W., Wang W. (2016). Vascularized bone grafting fixed by biodegradable magnesium screw for treating osteonecrosis of the femoral head. Biomaterials.

